# The Roles of Protein *S*-Palmitoylation in Cancers: From Dynamic Modulation to Therapeutic Potential

**DOI:** 10.34133/cancomm.0017

**Published:** 2026-04-02

**Authors:** Haonan Zheng, Xiaoyu Sun, Yang Gao, Qinbiao Wang, Jiaqi Wang, Minjie Wei, Yu Tang, Miao He

**Affiliations:** ^1^ Department of Pharmacology, School of Pharmacy, China Medical University, Shenyang, Liaoning, P. R. China.; ^2^ Liaoning Key Laboratory of Molecular Targeted Anti-Tumor Drug Development and Evaluation, Liaoning Cancer Immune Peptide Drug Engineering Technology Research Center, Shenyang, Liaoning, P. R. China.; ^3^ National Health Commission Key Laboratory of Advanced Reproductive Medicine and Fertility (China Medical University), Shenyang, Liaoning, P. R. China.; ^4^ Liaoning Medical Diagnosis and Treatment Center, Shenyang, Liaoning, P. R. China.; ^5^ Department of Oncology, Cancer Hospital of China Medical University, Liaoning Cancer Hospital and Institute, Shenyang, Liaoning, P. R. China.

## Abstract

Protein *S*-palmitoylation is a highly conserved posttranslational lipid modification that occurs on cysteine residues and critically influences protein maturation, subcellular localization, trafficking, and stability. Owing to its unique reversibility and dynamic nature, *S*-palmitoylation plays a pivotal role in cancer. This review comprehensively summarized the expression profiles and distribution of key cancer-related *S*-palmitoylation enzymes in recent years. Importantly, we highlight the specific mechanisms by which the dual states of palmitoylation and depalmitoylation function as a dynamic regulatory axis during the transformation of cancer cells into cancer stem cells and during the transition from a normal tissue environment to a tumor microenvironment. Furthermore, we discussed emerging therapeutic strategies targeting *S*-palmitoylation, including the development of specific inhibitors and competitive blockade of substrate-binding sites, which offer additional insights into the translational potential of *S*-palmitoylation as a therapeutic target for cancer.

## Introduction

Protein posttranslational modifications (PTMs) are critical regulatory mechanisms that control protein activity, stability, subcellular localization, and protein–protein interactions. Through protein PTMs, cells can rapidly respond to internal and external environmental changes and dynamically modulate signal transduction pathways, thereby influencing gene expression, cellular metabolism, and fate decisions [1–3].

Among these modifications, protein lipidation constitutes a major class that increases protein hydrophobicity and regulates the association of proteins with biological membranes and membrane microdomains. Protein lipidation mainly comprises long-chain fatty acylation, such as *N*-myristoylation and *S*-palmitoylation, isoprenoid-based prenylation, as well as other forms such as glycosylphosphatidylinositol anchoring and cholesterylation. Together, these lipid modifications regulate membrane targeting and the spatial organization of signaling proteins [4–6]. Within this group, protein palmitoylation represents a prototypical long-chain fatty acylation. Proteomic and functional studies indicate that thousands of proteins are palmitoylated and participate in diverse processes, including signal transduction, vesicle trafficking, cytoskeletal remodeling, and immune regulation, with broad implications for cancer biology [7,8]. The reversible attachment of long-chain fatty acids via thioester bonds locally increases protein hydrophobicity and provides a reversible lipid anchor, thereby regulating membrane association, stability, trafficking, protein–protein interactions, and downstream signaling.

Palmitoylation can be classified into 3 types: *S*-palmitoylation, *N*-palmitoylation, and *O*-palmitoylation. The similarities and differences among the 3 distinct forms of palmitoylation are summarized in Table 1. In this review, we focus on the reversible modification of cysteine residues by long-chain fatty acids through thioester bonds, commonly referred to as *S*-palmitoylation. This dynamic lipidation increases local hydrophobicity and provides a reversible membrane anchor that governs protein association with membranes, subcellular localization, stability, trafficking, and signaling output [9]. Chemically, cysteine *S*-palmitoylation is a prevalent form of long-chain protein *S*-acylation. However, most thioester-cleavage-based enrichment workflows, such as acyl–biotin exchange/acyl–resin-assisted capture/acyl–polyethylene glycol exchange, remove the thioester-linked acyl group during sample processing and therefore generally do not retain information on acyl-chain identity (palmitate versus other long-chain fatty acids) [10,11]. Unless otherwise stated, we used “*S*-palmitoylation” as shorthand for thioester-linked cysteine *S*-acylation and reserve this term for cysteine modifications only, not for *N*-palmitoylation or *O*-palmitoylation.

**Table 1.** Comparison of *S*-, *O*-, and *N*-palmitoylation

Feature	*S*-palmitoylation	*O*-palmitoylation	*N*-palmitoylation	Refs.
Chemical bond	Thioester bond (Cys-SH + palmitate)	Ester bond (Ser/Thr-OH + palmitoleate)	Amide bond (N-terminal Cys + palmitate)	[12,209,210]
Reversibility	Reversible (dynamic, labile)	Relatively stable; deacylation reported in specific contexts	Highly stable; no established dedicated “de-*N*-palmitoylation”; removal mainly via protein turnover	[18,209]
Major enzymes	Writers: DHHC *S*-acyltransferases (23 human DHHCs); erasers: APT1/2, ABHD17A to ABHD17C, PPTs	PORCN catalyzes Wnt *O*-acylation (commonly *O*-palmitoleoylation)	HHAT catalyzes amide-linked palmitate addition to the N-terminal cysteine of Hh ligands	[18,69,209–211]
Function	Regulates membrane association; controls trafficking/stability; modulates signal transduction (Wnt, Hippo, TGF-β, immune pathways); dynamic on–off regulation of signaling	Often required for ligand secretion and receptor engagement; key for Wnt morphogen activity/gradient formation	Confers strong hydrophobicity and membrane association of ligands; essential for Hh ligand activity/signaling range	[18,209,210]
Representative substrates	CD36, GLUT1, IFITM3, PD-L1, Ras, Src-family kinases, STAT3, TEAD	Wnt ligands (e.g., Wnt3A, Wnt5A)	Hh ligands (SHH/IHH/DHH)	[15,47,84,143,209,210,212–214]
Biological impact	Dynamic regulation of cancer progression, immune evasion, metabolism, trafficking	Essential for developmental signaling and gradient formation (Wnt)	Essential for developmental signaling (Hh)	[12,215,216]
Relevance to cancer	Strong and widely reported: CSCs, immune evasion, metastasis, metabolic adaptation	Impacts Wnt oncogenic pathways indirectly	Limited evidence; not a major regulatory mechanism in cancer	[12,217,218]

ABHD17A to ABHD17C, α/β hydrolase domain containing 17A to α/β hydrolase domain containing 17C; APT1/2, acyl–protein thioesterase 1/2; CD36, cluster of differentiation 36; CSC, cancer stem cell; Cys-SH, cysteine-sulfhydryl; DHHC, Asp–His–His–Cys motif; GLUT1, glucose transporter 1; Hh, Hedgehog; IFITM3, interferon-induced transmembrane protein 3; N-terminal Gly, N-terminal glycine; PD-L1, programmed death-ligand 1; PORCN, porcupine *O*-acyltransferase; PPT1, palmitoyl-protein thioesterase 1; Ras, rat sarcoma viral oncogene homolog; Ser/Thr-OH, serine/threonine-hydroxyl; SNAP-25 family, synaptosomal-associated protein 25 family; STAT3, signal transducer and activator of transcription 3; TEAD, transcriptional enhanced associate domain transcription factors; TGF-β, transforming growth factor-β; Wnt, Wingless/Integrated; Wnt3A, Wnt family member 3A; SHH, Sonic hedgehog; IHH, Indian hedgehog; DHH, Desert hedgehog.

Dysregulated *S*-palmitoylation has been linked to multiple hallmarks of cancer, including aberrant oncogenic signaling, cancer stemness, remodeling of tumor microenvironment (TME), and therapeutic resistance [12–17]. As a unique and reversible lipid modification, *S*-palmitoylation provides a tunable switch that integrates cancer-cell-intrinsic programs with extrinsic cues from the TME. In this review, we summarized recent advances in *S*-palmitoylation in cancer, charted its functional landscape across different cancer contexts, and highlighted its dynamic regulatory roles in cancer stem cells (CSCs), the TME, and oncogenic signaling networks. We also discussed the opportunities and challenges of targeting palmitoylation for cancer therapy, aiming to provide a concise framework for future mechanistic studies and translational strategies.

## Enzymes Involved in the Regulation of Protein *S*-Palmitoylation

**Fig. 1.**
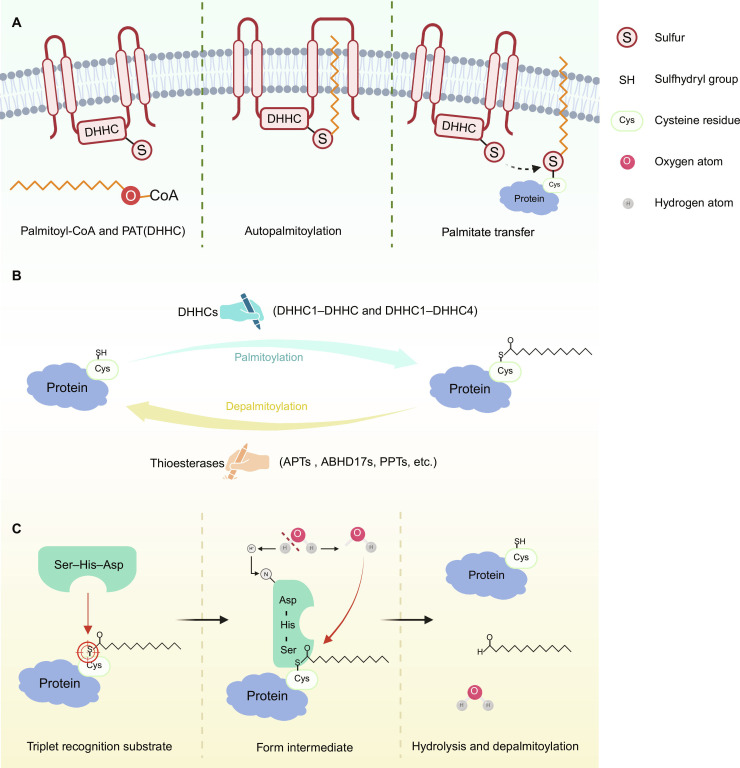
Dynamic protein *S*-palmitoylation process. (A) Protein *S*-palmitoylation catalyzed by Asp–His–His–Cys motif (DHHC) enzymes occurs in 3 steps: The DHHC enzyme cysteine (Cys) undergoes autoacylation with acyl–coenzyme A (CoA); the acyl group is then transferred to the substrate protein’s cysteine; and, in the third step, cysteine sites in proteins is identified, and *S*-palmitoylation modification is performed. (B) The dynamic and reversible nature of *S*-palmitoylation regulates protein localization and function. Palmitoyltransferase includes DHHCs (DHHC1 to DHHC9 and DHHC11 to DHHC24), which mediate the *S*-palmitoylation process. Thioesterases, including the acyl–protein thioesterase (APT), ABHD17, and palmitoyl–protein thioesterase (PPT) families, mediate depalmitoylation. (C) Depalmitoylation is catalyzed by a Ser–His–Asp catalytic triad. After substrate recognition, the triad activates the serine nucleophile, enabling it to attack the thioester bond linking palmitate to the cysteine residue, thereby forming an acyl–enzyme intermediate. This intermediate is subsequently hydrolyzed by water, releasing free palmitate and regenerating the active enzyme, while producing the deacylated protein with a restored cysteine sulfhydryl (Cys-SH). Illustration was created using BioRender.com. PAT, palmitoyl acyltransferase.

Protein *S*-palmitoylation is primarily catalyzed by the palmitoyl acyltransferase (PAT) family, while depalmitoylases, including thioesterases and membrane-associated serine hydrolases, mediate depalmitoylation [18–21]. Together, these enzymes constitute a unique and reversible modification cycle (Fig. 1). Evidence from the SwissPalm database (https://swisspalm.org/) indicates that more than 2,400 mammalian proteins undergo palmitoylation, as confirmed in over 100 proteomic and biochemical studies [22]. To date, over 150 cancer-associated proteins have been reported to be palmitoylated [23]. As a result, targeting palmitoylation and its associated enzymes has emerged as a promising direction in cancer research.

### Protein *S*-palmitoylation-related enzymes

*S*-palmitoyl transferases are a subset of PAT enzymes that catalyze *S*-palmitoylation. In cancers, these enzymes are implicated in driving tumor progression by promoting proliferation, modulating signaling cascades, altering membrane localization of oncogenic proteins, and affecting interactions between cancer cells and their microenvironment [12,14,24]. Therefore, *S*-palmitoyl transferases are considered as potential therapeutic targets, and the development of selective inhibitors may provide new avenues for cancer treatment.

#### Structure and classification of Asp–His–His–Cys palmitoyltransferase

Asp–His–His–Cys (DHHC) enzymes are a family of PATs that catalyze the reversible addition of palmitic acid to cysteine residue, a modification essential for protein localization, stability, and signaling [25]. Their defining hallmark is the highly conserved DHHC motif, which functions as the core catalytic domain necessary for palmitoyl transferase activity [20]. Early biochemical studies in yeast first identified DHHC-cysteine-rich domain (CRD) proteins such as palmitoyltransferase AKR1 (Akr1p) as bona fide palmitoyltransferases and demonstrated that an intact DHHC motif is required for catalytic function [26]. The DHHC motif is embedded within a ∼50-amino-acid CRD that coordinates zinc and is conserved from yeast to mammals, providing a structural scaffold that supports proper enzyme folding and activity [25,27]. Although additional domains contribute to membrane localization and substrate specificity, the DHHC motif itself represents the indispensable catalytic signature that functionally defines this enzyme family.

All DHHC enzymes share zinc-finger-like structural features and exert their critical functions through this structure. Structural studies confirm that the zinc finger domain is essential for maintaining the 3-dimensional conformation of DHHC proteins, particularly surrounding the catalytic site [25]. Although zinc ions do not participate directly in the catalytic reaction, they stabilize the region, ensuring the precise positioning of the active-site cysteine for catalysis. DHHC enzymes catalyze *S*-palmitoylation via a 3-step mechanism [25,28,29]. In the first step, the catalytic cysteine undergoes autoacylation by acyl–coenzyme A (CoA); in the second step, the acyl group is transferred to a cysteine residue on the substrate protein; and, in the third step, cysteine sites in proteins are identified, and *S*-palmitoylation modification is performed (Fig. 1A).

The human genome encodes 23 members of the DHHC family (i.e., DHHC1 to DHHC24, excluding DHHC10), which can be grouped into several subfamilies based on amino acid sequence homology and conserved DHHC motifs (Fig. 1B) [30]. Despite differences in chromosomal location, these enzymes share DHHC-CRD, which is essential for catalytic activity and plays a pivotal role in mediating *S*-palmitoylation [19].

Although the complete structural landscape of DHHC enzymes remains unresolved, recent advances using x-ray crystallography and cryo-electron microscopy have elucidated the structures of selected DHHC members, such as DHHC13 and DHHC17 [25]. These findings revealed substrate recognition mechanisms and the molecular basis of catalytic activity, thus deepening our understanding of the functional relevance of *S*-palmitoylation in membrane organization, signal transduction, and disease.

#### Functions of DHHC palmitoyltransferases

Enzyme localization dictates substrate accessibility and functional output. For example, DHHC1, DHHC2, DHHC5, DHHC12, and DHHC13 are predominantly localized to the plasma membrane, where they regulate receptor dynamics and cell signaling [31]. Enzymes, such as DHHC3 and DHHC9, locate in the endoplasmic reticulum (ER) or Golgi apparatus, primarily control protein maturation, folding, and vesicular trafficking [22,31]. Recent structural and biochemical studies further revealed that the transmembrane domains and active site architecture of DHHC enzymes contribute to their spatial distribution and substrate recognition [32,33]. As *S*-palmitoylation modulates protein–membrane interactions and trafficking, the precise positioning of each DHHC isoform determines substrate selectivity and spatiotemporal regulation of downstream signaling pathways [33,34]. Moreover, specific DHHC enzymes have been shown to selectively palmitoylate immuno-regulatory proteins based on their subcellular localization, reinforcing the functional relevance of compartmentalized activity [35].

The subcellular distribution of DHHC enzymes holds important functional importance in cancer. The spatial confinement of DHHCs governs their access to oncogenic or tumor-suppressive substrates, thereby directly influencing tumor progression. For example, DHHC9 preferentially palmitoylates the neuroblastoma RAS (NRAS) [36], a modification that enhances NRAS membrane localization and oncogenic signaling. Understanding such localization–function relationships has profound implications for the development of targeted cancer interventions. However, identifying universal rules governing DHHC substrate recognition and localization remains an unresolved challenge. Functionally, DHHC enzymes exhibit dual roles in cancer. Several members, including DHHC1, DHHC7, DHHC13, and DHHC22, have been shown to have tumor-suppressive functions [37–45]. In contrast, others, such as DHHC2, DHHC3, DHHC4, DHHC5, DHHC6, DHHC8, DHHC9, DHHC11, DHHC12, DHHC15, DHHC17, DHHC18, DHHC19, DHHC23, and DHHC24, are associated with oncogenic activity in a variety of cancer types and cellular contexts [46–63].

### Protein depalmitoylases

#### Structure and classification

Depalmitoylation is catalyzed by several thioesterase families, most of which are α/β-hydrolase-fold serine hydrolases, including acyl–protein thioesterases (APTs), such as APT1 and APT2 (also known as lysophospholipase 1 [LYPLA1] and LYPLA2) [64]; α/β hydrolase domain-containing (ABHD) proteins, such as ABHD10, ABHD12, and ABHD17A/B/C [65]; and the lysosomal depalmitoylase palmitoyl-protein thioesterase 1 (PPT1) [66] (Fig. 1B). Structures of APT1/2, PPT1, and ABHD10 have been resolved, and all of them share a conserved Ser–His–Asp catalytic triad [67]. These enzymes exhibit depalmitoylating activity in vitro and probably localize in the cytoplasm to enable dynamic regulation of *S*-palmitoylation.

#### The function of depalmitoylases

Depalmitoylases remove palmitoyl groups from cysteine residues, thereby regulating protein membrane association, trafficking, and function. Dysregulation of this modification is linked to tumor initiation and progression. In coordination with DHHC enzymes, depalmitoylases help maintain the dynamic nature of *S*-palmitoylation, which is essential for cellular signaling and homeostasis.

APT enzymes indirectly regulate key biological processes, including cell proliferation, differentiation, and migration, by modulating the *S*-palmitoylation status of critical signaling proteins [67,68]. ABHD17 family members also influence lipid metabolism and signal transduction by depalmitoylating specific protein substrates. The enzymatic activity is crucial for maintaining protein localization and cellular function [21,69]. Disruption of these enzymes has been implicated in oncogenesis, where altered *S*-palmitoylation can facilitate tumor growth and metastasis [21]. Mechanistically, depalmitoylation involves the catalytic triad, which activates the serine residue to attack the thioester bond, forming an acyl–enzyme intermediate that is subsequently hydrolyzed, releasing free palmitate and the deacylated protein (Fig. 1C) [70].

Unlike cytosolic depalmitoylases, PPT1 is a soluble lysosomal thioesterase that removes thioester-linked fatty acids (often palmitate) from *S*-palmitoylated proteins during lysosomal protein degradation, thereby facilitating their turnover/clearance [71]. In addition to being reported in association with cancer [72–74], PPT1 dysfunction has been linked to various neurodegenerative diseases [75,76]. Loss of PPT1 activity impairs lysosomal degradation, resulting in pathological lipid accumulation in the brain and other tissues. Notably, DHHC are predominantly localized to the ER/Golgi system, whereas lysosomal depalmitoylation is mainly attributed to the luminal thioesterase PPT1 during lysosomal turnover of *S*-acylated proteins. This compartmentalization may help coordinate *S*-palmitoylated protein degradation and the recycling of long-chain fatty acids [18].

Interestingly, current studies suggest that depalmitoylases are more abundantly expressed than DHHC enzymes [77,78], possibly ensuring timely reversal of *S*-palmitoylation events. The interplay between DHHCs and depalmitoylases underpins the reversible and tightly regulated nature of *S*-palmitoylation. In coordination with DHHC enzymes, depalmitoylases help maintain the dynamic balance of palmitoylation, which is essential for cellular signaling and homeostasis.

## The Role of Protein *S*-Palmitoylation in Cancer Stemness

CSCs are a major research focus in cancer initiation, progression, metastasis, and therapeutic resistance [79]. *S*-palmitoylation contributes to organ development and tissue homeostasis by regulating protein trafficking and epithelial polarity, and its dynamic regulation supports cellular adaptation to environmental stress, such as nutrient deprivation, through pathways such as autophagy [19,42,80]. By increasing the local hydrophobicity of proteins and providing a reversible lipid anchor, this modification modulates signal transduction, determines cell fate, and shapes cellular behavior under pathological conditions, thereby exerting a profound influence on oncogenesis and the functional maintenance of CSCs [8].

**Fig. 2.**
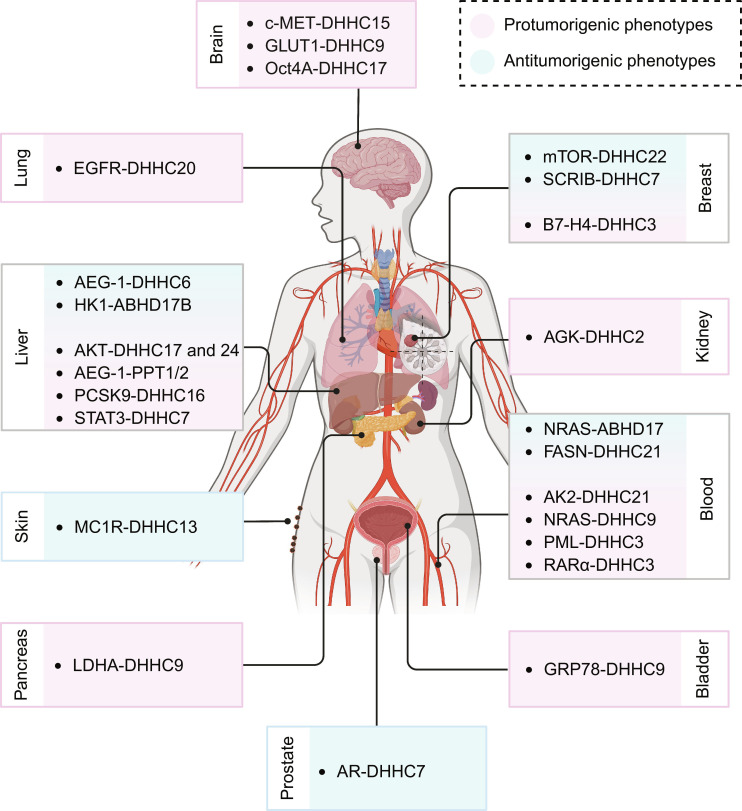
The bidirectional nature of *S*-palmitoylation in regulating cancer progression. *S*-palmitoylation regulators (e.g., Asp–His–His–Cys motifs [DHHCs], APTs, PPTs, and α/β hydrolase domain-containing [ABHDs]) act on different substrates across tissues from various organ, promoting or suppressing tumor progression in a context-dependent manner. Pink indicates axes reported to enhance malignant phenotypes (e.g., tumor growth, invasion/metastasis, immune evasion, or therapy resistance), whereas blue indicates axes reported to attenuate malignant phenotypes or increase therapy sensitivity, as defined in the cited studies. Illustration was created using BioRender.com. AEG-1, astrocyte elevated gene-1; AGK, acylglycerol kinase; AK2, adenylate kinase 2; AKT, AKT serine/threonine kinase; AMPK, AMP-activated protein kinase; AR, androgen receptor; B7-H4, B7 homolog 4; c-MET, MET proto-oncogene, receptor tyrosine kinase; EGFR, epidermal growth factor receptor; GLUT1, glucose transporter 1; GRP78, glucose-regulated protein 78 kDa; HK1, hexokinase 1; LDHA, lactate dehydrogenase A; mTOR, mechanistic target of rapamycin; NRAS, neuroblastoma RAS viral oncogene homolog; Oct4A, octamer-binding transcription factor 4A; PCSK9, proprotein convertase subtilisin/kexin type 9; PML, promyelocytic leukemia protein; PPT, palmitoyl-protein thioesterase; RARα, retinoic acid receptor α; SCRIB, Scribble cell polarity protein; STAT3, signal transducer and activator of transcription 3.

Protein *S*-palmitoylation is dysregulated across a wide range of human malignancies, including cancers of the digestive, respiratory, hematopoietic, urinary, reproductive, and nervous systems (Fig. 2) [46,81–83]. By conferring a reversible lipid anchor on key signaling and metabolic proteins, *S*-palmitoylation modulates their membrane association, stability, and activity, thereby influencing cancer cell proliferation, survival, invasion, and therapeutic response. Consistent with this central regulatory position, accumulating studies have revealed that protein *S*-palmitoylation can exert both pro- and antitumorigenic effects in a context-dependent manner. For example, DHHC9-mediated *S*-palmitoylation of glucose transporter 1 (GLUT1) promotes glycolysis and drives tumorigenesis in glioblastoma [84]; DHHC18-induced *S*-palmitoylation of malate dehydrogenase 2 enhances mitochondrial respiration and accelerates ovarian cancer cell proliferation [85]. Conversely, DHHC6-mediated *S*-palmitoylation of astrocyte elevated gene-1 (AEG-1) promotes its proteasomal degradation and inhibits hepatocellular carcinoma progression [86]; DHHC7-dependent *S*-palmitoylation of Scribble planar cell polarity protein enhances its tumor-suppressive activity in breast and ovarian cancers [42]; and *S*-palmitoylation of fatty acid synthase (FASN) by DHHC21 reduces FASN stability and lipid biosynthesis, thereby restraining diffuse large B cell lymphoma growth [87]. These examples underscore the substrate- and tissue-specific duality of protein *S*-palmitoylation in cancer and provide a conceptual basis for its roles in cancer stemness, discussed in the following subsections. A summary of the examples is presented in Table 2.

**Table 2.** The impact of dynamic *S*-palmitoylation on tumor progression

Role of dynamic palmitoylation	Cancer type	Enzymes	Mechanisms or effects	Refs.
Tumor-promoting	Glioblastoma	DHHC9; DHHC17; DHHC15	*S*-palmitoylation of GLUT1 maintains its plasma membrane localization, enhancing glucose uptake; concurrently, *S*-palmitoylation protects Oct4A from lysosomal degradation, preserving stemness factor stability and promoting tumor aggressiveness; *S*-palmitoylation of c-MET by DHHC15 drives glioblastoma stem cell tumorigenicity.	[62,84,219]
	Ovarian cancer	DHHC18	*S*-palmitoylation of mitochondrial respiratory chain components sustains oxidative phosphorylation, meeting high metabolic demands and driving malignant progression.	[85]
	Liver tumor	DHHC17 and DHHC24; DHHC16	*S*-palmitoylation regulates membrane localization and activity of target proteins; it increases PCSK9 activity, potentially influencing cholesterol metabolism and tumor growth.	[58,207]
	Hepatocellular carcinoma	DHHC7; PPT1/2	*S*-palmitoylation enhances STAT3 target gene expression; AEG-1 depalmitoylation might confer nonbeneficial effect on the treatment of HCC.	[16,86]
	Acute myeloid leukemia	DHHC9; DHHC21	Maintains NRAS *S*-palmitoylation and plasma membrane localization, sustaining oncogenic RAS signaling; regulates oxidative phosphorylation and the NRAS *S*-palmitoylation cycle to enhance leukemic cell proliferation.	[36,220]
	Acute promyelocytic leukemia	DHHC3	Palmitoyltransferase DHHC3 is essential for the oncogenic activity of PML/RARα in acute promyelocytic leukemia.	[221]
	Colorectal cancer	NA	Palmitic acid promotes *S*-palmitoylation of β-catenin at Cys466, blocking CK1/GSK-3-mediated phosphorylation and β-TrCP-dependent proteasomal degradation, thereby stabilizing β-catenin and promoting Wnt signaling.	[222]
	Renal cell carcinoma	DHHC2	*S*-palmitoylation maintains AGK at the plasma membrane, supporting its oncogenic functions.	[46]
	Pancreatic cancer	DHHC9; DHHC20	*S*-palmitoylation enhances LDHA activity to promote glycolysis; it inhibits YTHDF3 degradation, stabilizing RNA-binding protein function and supporting tumor progression.	[14,223]
	Bladder cancer	DHHC9	*S*-palmitoylation stabilizes Bip/GRP78 and retains it in the ER, supporting ER function and tumor cell survival.	[82]
	Lung caner	DHHC20	DHHC20-mediated EGFR *S*-palmitoylation sustains PI3K signaling; inhibition of this modification suppresses PI3K activity and reduces tumor growth in mutant KRAS lung cancer.	[224]
Tumor-suppressing	Breast cancer	DHHC22; DHHC7	Loss of DHHC22-mediated *S*-palmitoylation reduces mTOR stability, impairing growth signaling and suppressing tumor progression; DHHC7-mediated *S*-palmitoylation of Scribble regulates cell polarity.	[42,45]
	Hepatocellular carcinoma	DHHC6; ABHD17B	*S*-palmitoylation adversely regulates the stability of AEG-1, and *S*-palmitoylation might confer beneficial effect on the treatment of HCC; inhibition of HK1 *S*-palmitoylation attenuates HK1 release, altering tumor metabolism.	[86,117]
	Diffuse large B cell lymphoma	DHHC21	Loss of FASN *S*-palmitoylation decreases FASN stability and fatty acid synthesis, limiting tumor cell proliferation.	[87]
	Prostate cancer	DHHC7	DHHC7 depletion enhances oncogenic traits of prostate cancer cells	[83]
	Melanoma	DHHC13	*S*-palmitoylation of MC1R suppresses UVB-induced transformation of human melanocytes, acting as a tumor-suppressive mechanism.	[43]
	Acute myeloid leukemia	ABHD17	ABHD17 regulation of plasma membrane palmitoylation and NRas-dependent cancer growth.	[21]

AEG-1, astrocyte elevated gene-1; AGK, acylglycerol kinase; BiP, binding immunoglobulin protein; CK1, casein kinase 1; c-MET, MET proto-oncogene, receptor tyrosine kinase; EGFR, epidermal growth factor receptor; ER, endoplasmic reticulum; FASN, fatty acid synthase; GLUT1, glucose transporter 1; GRP78, glucose-regulated protein 78; GSK-3, glycogen synthase kinase 3; HK1, hexokinase 1; KRAS, Kirsten rat sarcoma viral oncogene homolog; LDHA, lactate dehydrogenase A; MC1R, melanocortin 1 receptor; mTOR, mammalian target of rapamycin; NA, not available; NRAS, neuroblastoma RAS viral oncogene homolog; Oct4A, octamer-binding transcription factor 4A; PCSK9, proprotein convertase subtilisin/kexin type 9; PI3K, phosphatidylinositol 3-kinase; PPT1/2, palmitoyl-protein thioesterase 1/2; RAS, rat sarcoma viral oncogene homolog; SND1, staphylococcal nuclease and tudor domain containing 1; STAT3, signal transducer and activator of transcription 3; UVB, ultraviolet B; Wnt signaling, Wingless/Int1 signaling pathway; Wnt1, Wingless-Int1; YTHDF3, YTH *N*^6^-methyladenosine RNA binding protein F3; β-TrCP, β-transducin repeat-containing E3 ubiquitin protein ligase.

Accumulating evidence indicates that the Wingless/Int-1 (Wnt), Hippo, transforming growth factor-β (TGF-β), and Hedgehog (Hh) signaling pathways play a key role in CSCs’ self-renewal, survival, and cellular plasticity [88]. These pathways are also essential during embryonic development, organogenesis, and adult tissue repair. Dysregulation of these pathways, whether by aberrant activation or suppression, is strongly implicated in the onset and progression of multiple cancer types. *S*-palmitoylation modulates the activity of these signaling axes by altering the lipophilicity, subcellular localization, and stability of core signaling proteins.

### Protein *S*-palmitoylation in the Wnt/β-catenin pathway

The Wnt signaling pathway comprises essential components, including Wnt ligands, Frizzled receptors, low-density lipoprotein receptor-related protein 5/6 (LRP5/6) co-receptors, Dishevelled proteins, β-catenin, and T cell factor/lymphoid enhancer-binding factor (TCF/LEF) transcription factors. The pathway is a key regulator of cell proliferation, differentiation, and tissue homeostasis and plays a central role in embryogenesis and tissue regeneration [89,90].

**Fig. 3.**
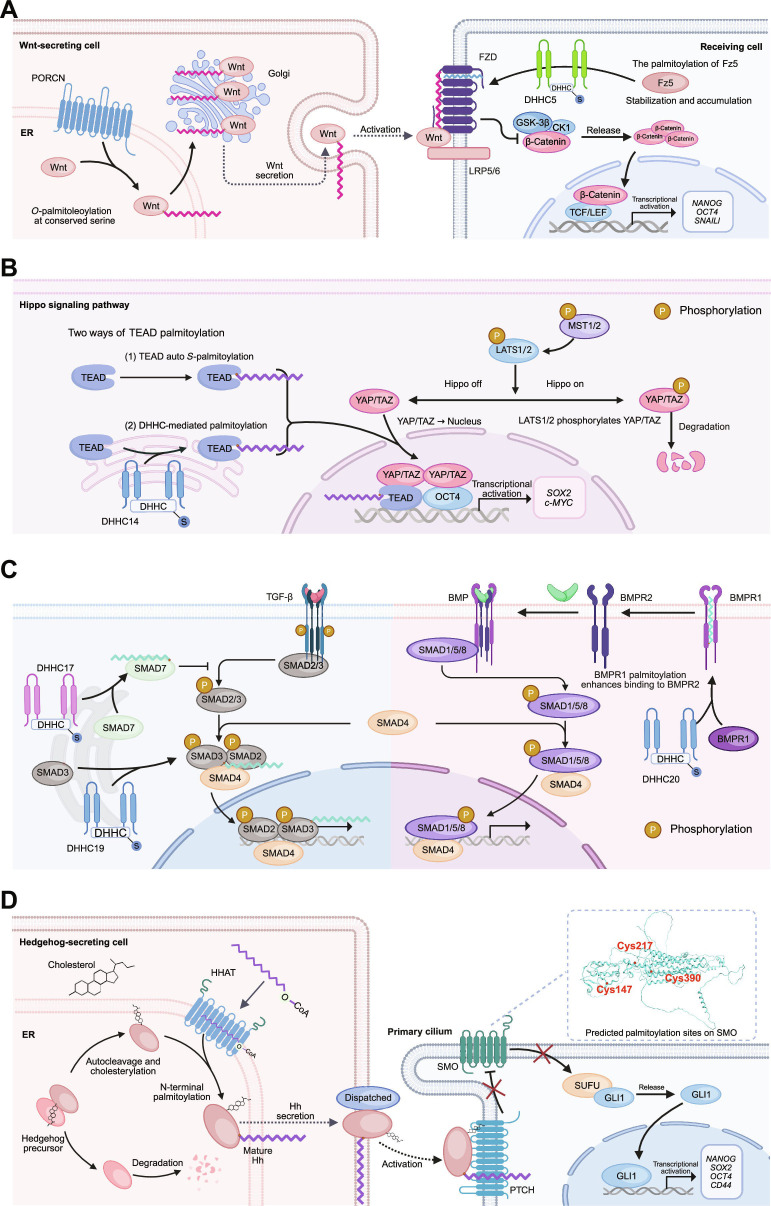
Regulation of tumor-stemness-pathway-related genes by palmitoylation. (A) Left: Wingless/Integrated (Wnt) ligands are modified in the secretory pathway by porcupine *O*-acyltransferase (PORCN)–catalyzed *O*-acylation (often *O*-palmitoleoylation) at a conserved serine, which is required for efficient trafficking through the Golgi apparatus (Golgi), secretion, and bioactivity. Right: Secreted acylated Wnt binds the Frizzled (FZD)–low-density lipoprotein receptor-related protein 5/6 (LRP5/6) receptor complex to initiate signaling, leading to inhibition of the β-catenin destruction complex, β-catenin stabilization/accumulation, and nuclear T cell factor/lymphoid enhancer factor (TCF/LEF)-dependent transcription. In parallel, Asp–His–His–Cys motif 5 (DHHC5)-mediated *S*-palmitoylation of frizzled class receptor 5 (Fz5) promotes its endoplasmic reticulum (ER)-to-Golgi trafficking and receptor maturation, thereby enhancing membrane localization and signaling output. (B) When Hippo signaling is on, the mammalian sterile 20-like kinase 1/2 (MST1/2)–large tumor suppressor kinase 1/2 (LATS1/2) cascade phosphorylates Yes-associated protein/transcriptional coactivator with PDZ-binding motif (YAP/TAZ) (P), promoting cytoplasmic retention and degradation. When Hippo signaling is off, dephosphorylated YAP/TAZ translocates into the nucleus and cooperates with transcriptional enhanced associate domain (TEAD) (and OCT4) to activate transcriptional programs associated with proliferation and stemness (e.g., *SOX2* and *c-MYC*). TEAD palmitoylation supports TEAD–YAP/TAZ complex formation and transcriptional output. The schematic illustrates 2 routes for TEAD palmitoylation: (1) TEAD auto-*S*-palmitoylation and (2) DHHC-mediated palmitoylation (shown here as DHHC14), both converging on enhanced TEAD hydrophobicity/stability and strengthened YAP/TAZ binding. P, phosphorylation. (C) Left: DHHC17-mediated *S*-palmitoylation of SMA- and MAD-related protein (SMAD) family member 7 (SMAD7) promotes its membrane recruitment/anchoring, thereby enhancing its inhibitory function and restraining transforming growth factor-β (TGF-β) signal propagation. In contrast, DHHC19-mediated *S*-palmitoylation of SMAD3 increases its membrane stability and sustains downstream SMAD transcriptional output. Right: DHHC20-catalyzed *S*-palmitoylation of bone morphogenetic protein (BMP) receptor type 1 (BMPR1) at membrane-proximal cysteines enhances receptor membrane localization and/or BMPR1–BMPR2 complex formation, facilitating SMAD1/5/8 phosphorylation, SMAD4 complex assembly, and nuclear signaling. (D) Hedgehog (Hh) precursors undergo Hh-C–mediated autoprocessing to generate the N-terminal signaling fragment (Hh-N) and enable C-terminal cholesterylation of Hh-N. Hh acyltransferase (HHAT) further catalyzes amide-linked *N*-palmitoylation at the N-terminal cysteine, producing a lipid-modified mature Hh ligand that is secreted (Dispatched) and engages Patched (PTCH) in receiving cells. Hh binding relieves PTCH-mediated repression of Smoothened (SMO) at the primary cilium, promoting dissociation of *GLI* from suppressor of fused (SUFU) and activation of *GLI*-dependent transcription (e.g., *NANOG*, *SOX2*, *OCT4*, and *CD44*). The inset highlights in-silico-predicted putative *S*-palmitoylation sites on SMO (Cys147, Cys217, and Cys390), which require experimental validation. Red X marks PTCH-mediated repression of SMO in the absence of Hh ligand; ligand binding relieves this repression to enable SMO activation. Illustration was created using BioRender.com. CD44, cluster of differentiation 44; CK1, casein kinase 1; c-MYC, cellular MYC; CoA, coenzyme A; GLI1, *GLI* family zinc finger 1; GSK-3β, glycogen synthase kinase 3β; NANOG, Nanog homeobox; Oct4, octamer-binding transcription factor 4; SARA, SMAD anchor for receptor activation; Smurf2, SMAD ubiquitination regulatory factor 2; SNAIL, Snail family zinc finger; SOX2, SRY-box transcription factor 2.

Effective Wnt signaling is critically dependent on palmitoylation of Wnt ligands. Prior to secretion, Wnt proteins undergo *O*-palmitoylation by the membrane-bound *O*-acyltransferase porcupine (PORCN), a modification essential for their bioactivity and efficient secretion [91,92]. Palmitoylated Wnt ligands exhibit enhanced binding to Frizzled receptors and LRP5/6 co-receptors, thereby amplifying downstream signaling. In receiving cells, studies have shown that the Frizzled-5 (Fz5) receptor also requires *S*-palmitoylation to facilitate ER to Golgi trafficking, a prerequisite for receptor maturation and function (Fig. 3A). Zheng et al. [93] first identified the *S*-palmitoylation site on Fz5 and demonstrated its necessity for ER–Golgi transport. Subsequent work by Teo’s group [94] revealed that DHHC5 mediates this *S*-palmitoylation, enhancing Fz5 membrane localization and signaling capacity, thereby increasing Wnt signaling efficiency in cancer cells.

Signal transduction is initiated by the binding of Wnt ligands to Frizzled receptors and LRP5/6 co-receptors. Frizzled activation propagates the signal via Dishevelled, which, in turn, inhibits glycogen synthase kinase 3β (GSK-3β) and casein kinase 1 (CK1), resulting in the stabilization and accumulation of β-catenin. Once translocated to the nucleus, β-catenin associates with TCF/LEF transcription factors to activate downstream target genes, thereby modulating self-renewal and differentiation in CSCs (Fig. 3A) [89].

In CSCs, it has been demonstrated to play a pivotal role in cancer recurrence and metastasis by sustaining stem-like traits. For example, Transmembrane 4 L6 family member 1 enhances CSCs properties by activating the Wnt/β-catenin/cellular myelocytomatosis oncogene (*c-MYC*)/SRY-box transcription factor 2 (*SOX2*) axis during colorectal cancer recurrence [95]. Abnormal Wnt pathway activation is also reported in thyroid, liver, and cervical cancers, where it facilitates malignant progression by reinforcing CSCs’ self-renewal and invasiveness [95–99].

Notably, cysteine *S*-palmitoylation is characterized by a labile thioester linkage and enzyme-regulated acylation–deacylation cycling. DHHC5-mediated *S*-palmitoylation of Fz5 substantially contributes to its membrane stability and signaling efficacy [94]. These findings underscore the multilayered regulatory roles of palmitoylation in Wnt signaling, particularly in sustaining CSCs traits and enhancing oncogenic signaling during cancer progression [92–94].

### Protein *S*-palmitoylation in the Hippo pathway

The Hippo signaling pathway is an evolutionarily conserved regulatory network initially identified in *Drosophila* [100]. In mammals, the pathway exerts its function by promoting the sequential phosphorylation of mammalian sterile 20-like kinase 1/2 (MST1/2) and large tumor suppressor kinase 1/2 (LATS1/2), which, in turn, inhibit the nuclear localization of the downstream effectors Yes-associated protein and transcriptional coactivator with PDZ-binding motif (YAP/TAZ). Phosphorylated YAP/TAZ are retained in the cytoplasm, preventing the transcription of genes related to cell proliferation and survival. Upon Hippo pathway inactivation, unphosphorylated YAP/TAZ translocate to the nucleus, bind to transcriptional enhanced associate domain (TEAD) transcription factors, and induce the expression of genes that drive cell growth, inhibit apoptosis, and sustain stemness (Fig. 3B) [101–103]. Dysregulation of Hippo signaling is closely associated with the progression of various cancers, particularly through its role in maintaining CSC properties. Constitutive activation of YAP/TAZ not only endows differentiated somatic cells with stem-like features but also promotes CSC enrichment by regulating critical transcriptional targets such as *SOX2*, *SOX9*, and octamer-binding transcription factor 4 (*OCT4*). In addition, it enhances cancer cell invasiveness and chemoresistance [104–106]. These findings highlight the importance of the YAP/TAZ axis in cancer progression and suggest its potential as a therapeutic target for modulating cancer stemness.

Beyond YAP/TAZ regulation, other Hippo pathway components are subject to PTMs, among which *S*-palmitoylation plays a crucial role [107]. TEAD transcription factors contain specific *S*-palmitoylation sites, which increase their lipophilicity, facilitating their incorporation into lipid-rich environments such as the plasma membrane and enhancing their interaction with YAP to form a stable TEAD–YAP complex (Fig. 3B). In 2022, Sun and colleagues [108] demonstrated that *S*-palmitoylation substantially contributes to the structural stability and transcriptional activity of TEAD proteins. This modification alters the TEAD conformation, enhancing its affinity for YAP and recruitment of transcriptional machinery, thereby boosting transcriptional output. Notably, recent evidence suggests that TEAD palmitoylation may also be subject to context-dependent enzymatic control, for example, DHHC14 was identified as a key PAT promoting TEAD palmitoylation in an inflammatory renal setting (Fig. 3B) [109]. TEAD *S*-palmitoylation not only promotes its association with YAP/TAZ but also up-regulates a broad spectrum of oncogenic targets, including *SOX2* [110], *c-MYC* [108], and connective tissue growth factor (*CTGF*) [111]. For instance, *S*-palmitoylation-mediated activation of *SOX2* supports the self-renewal of CSCs, while *c-MYC* up-regulation accelerates cancer cell proliferation. These findings provide mechanistic insight into how Hippo pathway dysfunction contributes to malignant transformation. Importantly, TEAD *S*-palmitoylation also presents a therapeutic opportunity. Inhibition of TEAD *S*-palmitoylation markedly disrupts its interaction with YAP/TAZ, attenuates TEAD–YAP complex formation, and suppresses transcription of protumorigenic genes [108]. In consideration of the reversible nature of *S*-palmitoylation, this modification may serve as a dynamic switch regulating Hippo pathway output in response to microenvironmental changes, further emphasizing its regulatory complexity within TME.

### Protein *S*-palmitoylation in TGF-β and bone morphogenetic protein signaling

TGF-β and bone morphogenetic protein (BMP) signaling are 2 major branches of the TGF-β superfamily and are central regulators of cell fate decisions, including proliferation, differentiation, apoptosis, stem cell maintenance, and tissue homeostasis [112]. Despite the presence of shared core components, such as SMA- and MAD-related (SMAD) proteins, the 2 pathways exhibit distinct biological functions and regulatory contexts. TGF-β signaling is predominantly involved in immunity, fibrosis, and epithelial–mesenchymal transition (EMT) and can exert both tumor-suppressive and prometastatic effects within TME [113]. Conversely, BMP signaling primarily governs bone and cartilage development, embryonic patterning, and stem cell fate decisions during tissue regeneration [114]. Recent studies have shown that *S*-palmitoylation has a multifaceted regulatory effect on both the TGF-β and BMP pathways. By modulating the membrane localization, stability, and signaling efficacy of key pathway components, *S*-palmitoylation directly influences TGF-β activity and affects the ability of cancer cells to adapt to their surrounding microenvironment, thereby facilitating cancer progression and immune evasion.

As shown in Fig. 3C, TGF-β signaling primarily activates SMAD2/3, whereas BMP signaling activates SMAD1/5/8; phosphorylated SMADs form complexes with SMAD4 and translocate to the nucleus to regulate transcription. In TGF-β pathways, the membrane residency of inhibitory SMAD proteins such as SMAD7 is critical for their suppressive function [115]. Voytyuk et al. [112] reported that *S*-palmitoylation of SMAD7 at Cys415 and Cys417, catalyzed by DHHC17, induces its nuclear export and membrane translocation, thereby enhancing its inhibitory activity on TGF-β signaling. These findings demonstrate that *S*-palmitoylation restricts signal amplification during cancer progression by promoting membrane anchoring of inhibitory SMADs (Fig. 3C). In contrast, *S*-palmitoylation of SMAD3 facilitates TGF-β signaling and contributes to its tumor-promoting effects. Fan et al. [116] found that DHHC19-mediated *S*-palmitoylation increases SMAD3 membrane stability and prolongs its activation, thereby enhancing transcription of stromal markers and promoting glioblastoma cell invasion and chemoresistance (Fig. 3C). This reveals a potential positive feedback loop in TGF-β signaling, where *S*-palmitoylation amplifies protumorigenic SMAD activity. Beyond classical SMAD-mediated signaling, *S*-palmitoylation also contributes to the metabolic rewiring of cancer cells in response to TGF-β stimulation. In a liver fibrosis model, Chen et al. [117] demonstrated that TGF-β-induced *S*-palmitoylation of hexokinase 1 (HK1) promotes its membrane translocation and subsequent packaging into exosomes, which are then transferred to cancer cells. This process supports metabolic reprogramming and enhances cancer cell survival under nutrient-deprived conditions, offering hepatocellular carcinoma a competitive advantage in the microenvironment. In the BMP pathway, Li et al. [118] used a *Drosophila* model to show that *S*-palmitoylation promotes membrane localization of an inhibitory SMAD, thereby increasing the efficiency of BMP pathway suppression. In the BMP signaling cascade, receptor complex formation between BMP receptor type 1 (BMPR1) and BMPR2 is essential for pathway activation. *S*-palmitoylation of BMPR1 at multiple membrane-proximal cysteine residues, catalyzed by DHHC20, substantially enhances its membrane localization and stability. This modification is hypothesized to alter the conformation of BMPR1 or promote its association with lipid rafts, thereby improving its binding efficiency with BMPR2 (Fig. 3C) [119].

### Palmitoylation and other lipid modifications in the Hh pathway

The Hh signaling pathway is a pivotal developmental cascade that regulates embryogenesis and adult tissue homeostasis [120,121]. It was first identified in *Drosophila*; the pathway contributes broadly to organ development, cellular differentiation, and tissue repair [122]. Its core components include Hh ligands, such as Sonic hedgehog, the receptor Patched (PTCH), the signal transducer Smoothened (SMO), and downstream glioma-associated oncogene homolog (*GLI*) transcription factors.

Aberrant Sonic hedgehog signaling is frequently implicated in cancers among the 3 mammalian Hh ligands—Sonic, Indian, and Desert Hh [123]. Hh precursors undergo autocatalytic processing within the C-terminal domain (Hh-C), which cleaves the precursor into the N-terminal signaling fragment (Hh-N) and a Hh-C and enables maturation of Hh-N (Fig. 3D). This processing simultaneously enables lipidation of Hh-N, which is essential for its signaling competence. Specifically, Hh-N receives 2 key lipid modifications: Cholesterol is covalently attached to the C terminus of Hh-N, and Hh acyltransferase (HHAT) catalyzes the addition of a palmitoyl group to the N-terminal cysteine of Hh-N [124]. These modifications enhance the hydrophobicity, membrane affinity, and signaling capacity of Hh proteins, enabling efficient binding to PTCH and activation of downstream signaling cascades [125].

Upon Hh ligand binding, PTCH-mediated repression of SMO is relieved, allowing SMO to activate *GLI* transcription factors, particularly GLI1, which then induce the transcription of key pluripotency-associated genes such as Nanog homeobox (*NANOG*), *SOX2*, *OCT4*, and cluster of differentiation 44 (*CD44*) [126,127]. This activation reinforces CSCs’ properties, including self-renewal, proliferative potential, and multidrug resistance [126–128]. Furthermore, aberrant GLI1 activation is a hallmark of CSCs. For example, GLI1 can be transcriptionally up-regulated via recruitment of DNA topoisomerase I to its promoter by a circular RNA derived from importin 11 (*IPO11*), thereby amplifying Hh signaling and driving cancer progression [129,130].

SMO, a critical Hh pathway component, plays a central role in propagating the signal to *GLI* factors [131]. Although direct experimental evidence for SMO palmitoylation remains lacking, its strong membrane localization suggests that palmitoylation may occur under specific conditions. Using Molecular Operating Environment-based computational modeling, we predicted Cys147, Cys217, and Cys390 as putative *S*-palmitoylation sites (Fig. 3D). Such modifications could enhance SMO membrane anchoring and signaling efficiency, further promoting downstream *GLI* activation and expression of genes associated with cancer cell proliferation, differentiation, and CSC maintenance. Determining whether SMO undergoes palmitoylation in vivo remains an important question for fully understanding its functional regulation.

Palmitoylation also facilitates cross-talk between signaling pathways. For instance, GLI1 activity is not solely governed by Hh signaling but also intersects with Wnt/β-catenin signaling to reinforce CSC phenotypes. Degirmenci et al. demonstrated that GLI1-expressing mesenchymal cells establish a Wnt-secreting niche in the colon that sustains stem cell homeostasis [132]. Genetic ablation of Wnt secretion in these cells disrupts colonic stem cell populations, compromises epithelial integrity, and ultimately results in lethality in mice [132,133]. Palmitoylation enables efficient pathway cross-talk by enhancing ligand secretion, membrane association, and signal transduction. The integration of multiple prostemness pathways is facilitated by palmitoylation, which modulates signaling convergence nodes such as GLI1–β-catenin interaction.

## The Role of Protein *S*-Palmitoylation in the TME

The TME is a complex ecosystem comprising cancer cells, stromal components, vasculature, immune populations, extracellular matrix, hypoxic niches, and various signaling molecules, which collectively drive tumorigenesis, metastasis, therapeutic resistance, and maintenance of stemness in cancer [134]. CSCs within the TME dynamically interact with processes such as EMT, angiogenesis, hypoxia, and inflammation to sustain tumor growth and recurrence. Hypoxia induces metabolic reprogramming, such as the Warburg effect and lipid oxidation, and stabilizes hypoxia-inducible factors (HIFs) to activate adaptive transcriptional programs, promote persistent inflammation, and modulate immune functions [135]. These interconnected processes constitute a hypoxia–inflammation–immunity interrelated metabolic network that enables cancer cells to thrive under environmental stress. *S*-palmitoylation regulates key events within this network by enhancing HIF signaling via membrane anchoring of metabolic enzymes and transporters, promoting inflammasome assembly and cytokine release, and modulating immune checkpoint expression and antigen presentation. These regulatory functions make *S*-palmitoylation central to cancer cell adaptation, immune evasion, and stemness maintenance, providing a basis for further therapeutic exploration.

### The roles of hypoxia-induced protein *S*-palmitoylation in TME adaptation

Hypoxia, a hallmark of solid tumors, markedly influences cancer cell proliferation, invasion, metabolic adaptation, and therapeutic responsiveness [135]. Under oxygen-deprived conditions, cancer cells activate HIFs to induce the expression of genes involved in cell migration, matrix remodeling, and energy metabolism, thereby enhancing their ability to survive under environmental stress [136].

#### Mechanistic link between hypoxia and *S*-palmitoylation

**Fig. 4.**
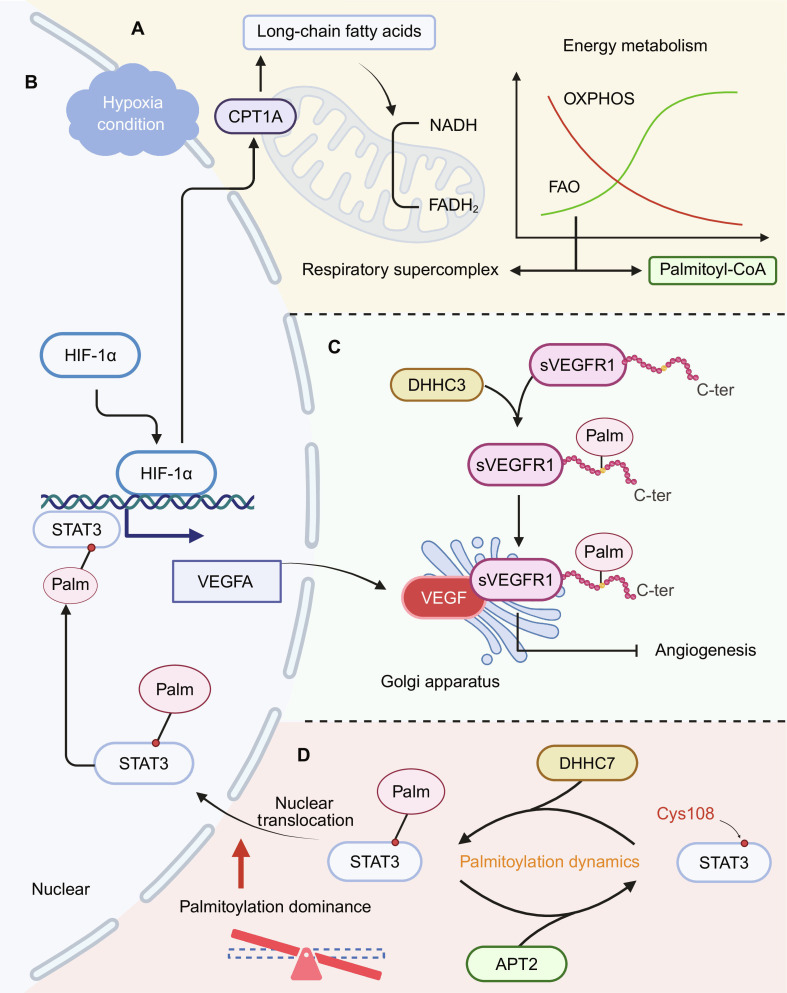
*S*-palmitoylation coordinates hypoxia adaptation in tumor cells through multiple mechanisms. (A) With suppressed oxidative phosphorylation (OXPHOS), fatty acid oxidation (FAO) compensates by producing reduced nicotinamide adenine dinucleotide (NADH), reduced flavin adenine dinucleotide (FADH_2_), and palmitoyl–CoA, supporting both energy supply and palmitoylation (Palm). Respiratory supercomplexes refer to higher-order assemblies of electron transport chain complexes that can improve respiratory efficiency; FAO has been reported to support the assembly of mitochondrial respiratory supercomplexes under hypoxic/metabolic stress. (B) Under hypoxia, hypoxia-inducible factor 1 subunit α (HIF-1α) is stabilized and translocated to the nucleus, where it up-regulates the expression of vascular endothelial growth factor A (VEGFA) and signal transducer and activator of transcription 3 (STAT3), and induces carnitine palmitoyltransferase 1A (CPT1A) to support FAO. (C) *S*-palmitoylation of soluble VEGF receptor 1 (VEGFR1) at its C terminus (C-ter) by DHHC3 promotes membrane anchoring and Golgi apparatus (Golgi) retention, enhancing VEGF binding and inhibiting angiogenesis. (D) STAT3 is dynamically palmitoylated at cysteine-108 (Cys108) by DHHC7 and acyl–protein thioesterase 2 (APT2), facilitating nuclear translocation and transcriptional activation in cooperation with HIF-1α. Illustration was created using BioRender.com.

In a hypoxic microenvironment, oxidative phosphorylation (OXPHOS) is compromised, and fatty acid oxidation (FAO) becomes the compensatory energy source for cells. FAO optimizes the assembly of mitochondrial respiratory supercomplexes, enhancing mitochondrial efficiency and adaptability (Fig. 4A) [137]. HIF-1α facilitates the import of long-chain fatty acids into mitochondria for β-oxidation by inducing the expression of carnitine palmitoyltransferase 1A (CPT1A), thereby generating reduced nicotinamide adenine dinucleotide (NADH) and reduced flavin adenine dinucleotide (FADH_2_) to fuel the electron transport chain (Fig. 4A and B) [137]. Simultaneously, adenosine monophosphate (AMP)-activated protein kinase (AMPK), an energy sensor, is activated under hypoxia or adenosine triphosphate (ATP) depletion. This enhances the activity of FAO enzymes, such as CPT1A and acyl–CoA dehydrogenase long chain, further promoting FAO flux [138]. This coordinated mechanism ensures ATP production under hypoxia and provides palmitoyl–CoA as a substrate for protein *S*-palmitoylation.

*S*-palmitoylation possibly enhances the stability and activity of FAO-associated enzymes under hypoxic stress, thereby promoting metabolic plasticity and improving cellular survival in energy-deprived environments. Whether *S*-palmitoylation directly participates in this structural reorganization remains unclear; however, these observations suggest that hypoxia-driven changes in lipid metabolism create a permissive landscape for *S*-palmitoylation to fine-tune mitochondrial function.

In addition to metabolic rewiring, hypoxia-induced stress engages in palmitoylation-dependent control of autophagy. DHHC7-mediated *S*-palmitoylation of autophagy-related protein 16-like 1 is necessary for its membrane recruitment, microtubule-associated protein 1 light chain 3 family lipidation, and efficient autophagosome formation, thereby functionally linking DHHC PATs to autophagy initiation [139]. *S*-palmitoylation of the multispanning membrane protein autophagy-related protein 9A coordinates its trafficking from the Golgi/endosomal compartments to phagophore formation sites, acting as a critical trigger for the onset of autophagy under stress conditions [140]. These reports delineate an “autophagy machinery palmitoylation network” that couples lipid modification to stress-adaptive autophagy in cancer cells under hypoxia and metabolic challenge.

*S*-palmitoylation also contributes to mitochondrial quality control. Under hypoxia, glycerophosphocholine phosphodiesterase 1 is depalmitoylated and translocated to the outer mitochondrial membrane, where it triggers voltage-dependent anion channel 1 ubiquitination and mitophagy. This process enables the clearance of damaged mitochondria and supports metabolic homeostasis [141]. Collectively, these reports highlight the importance of *S*-palmitoylation in preserving organelle integrity and facilitating metabolic adaptation.

#### *S*-palmitoylation-driven effects in the hypoxic TME

Hypoxia also induces the expression of vascular endothelial growth factor (VEGF), carbonic anhydrase IX, and GLUT1, further enhancing the metabolic adaptability of cancer cells and their capacity to remodel the microenvironment [135,136].

Notably, VEGF expression under hypoxia is not only regulated at the transcriptional level but also modulated posttranslationally via protein *S*-palmitoylation. DHHC3-mediated *S*-palmitoylation of the soluble VEGF receptor 1 (sVEGFR1; also known as sFlt-1) at its C terminus promotes its anchoring and stabilization in the Golgi membrane, thereby enhancing its affinity for VEGF, sequestering VEGF away from sVEGFR2, and reducing angiogenesis (Fig. 4C) [142]. In the absence of DHHC3 or upon pharmacological inhibition of *S*-palmitoylation, membrane-tethered VEGFR1 (or mFLT1) loses *S*-palmitoylation and shows increased lysosomal trafficking and reduced stability, thereby weakening its decoy restraint and favoring increased VEGFR2 signaling/angiogenic sprouting [142]. These findings suggest that *S*-palmitoylation is a key modulator of hypoxia-induced angiogenesis.

Signal transducer and activator of transcription 3 (STAT3), a HIF-associated transcriptional cofactor, is also regulated by *S*-palmitoylation. STAT3 is dynamically palmitoylated at Cys108 by DHHC7 and depalmitoylated by APT2, a cycle that enhances its nuclear translocation and DNA binding capacity, thereby enhancing transcriptional activity (Fig. 4D) [143]. Considering the coregulatory role of STAT3 and HIF-1α in hypoxia-responsive gene expression, this modification may further potentiate hypoxic signaling, reinforcing cellular adaptation under oxygen-deprived conditions (Fig. 4B).

### Regulatory roles of protein *S*-palmitoylation in the immune microenvironment

The tumor immune microenvironment sustains immune evasion through a network of synergistic mechanisms, including disruption of antigen recognition, shielding of innate immune sensing, intercellular diffusion of local and systemic inhibitory signals, and suppression of terminal effector functions [144–146]. Within this network, *S*-palmitoylation exerts critical regulatory effects at multiple levels by modulating the stability, trafficking, and signaling capacity of membrane-associated immune regulators [5,12,13,15,147].

In this section, we summarized the mechanisms by which *S*-palmitoylation promotes immune suppression through 4 interconnected modules that ultimately converge on T cell activation: (a) stabilization of immune checkpoint molecules, (b) interference with antigen presentation, (c) modulation of pattern recognition receptor (PRR) and innate immune sensing, and (d) regulation of exosome biogenesis and cargo sorting.

#### Stabilization of immune checkpoint molecules

Aberrant stabilization and persistent expression of immune checkpoint molecules are key strategies by which cancer cells evade immune surveillance [148,149]. Programmed death-ligand 1 (PD-L1) is a prototypical example: Its membrane stability and immunosuppressive capacity are governed by *S*-palmitoylation [15,35].

**Fig. 5.**
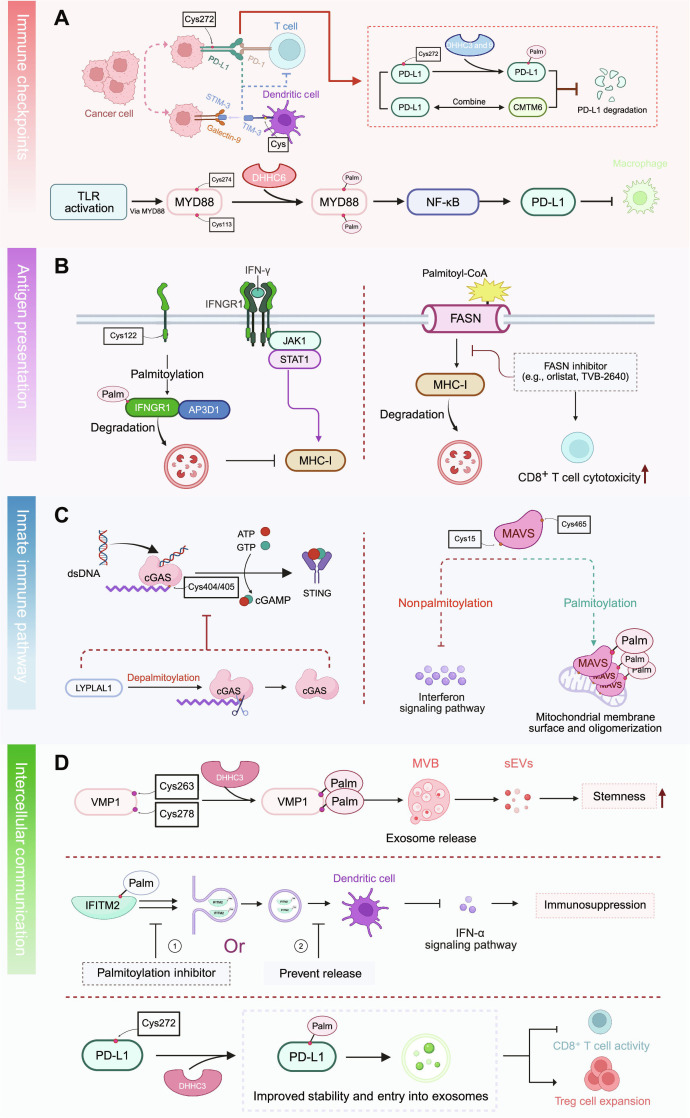
*S*-palmitoylation regulates tumor immune evasion at multiple levels in the tumor microenvironment. (A) *S*-palmitoylation of programmed death-ligand 1 (PD-L1) at cysteine-272 (Cys272) by Asp–His–His–Cys motif 3 (DHHC3) and DHHC9 prevents lysosomal degradation and promotes the interaction with CKLF-like MARVEL transmembrane-domain-containing 6 (CMTM6), stabilizing its membrane localization and enhancing immunosuppressive activity. Similar modifications of T cell immunoglobulin and mucin-domain-containing protein 3 (TIM3) and cluster of differentiation 47 (CD47) facilitate anchoring on dendritic cells and cancer cells, suppressing antigen presentation and phagocytosis. Palmitoylated myeloid differentiation primary response 88 (MYD88) activates nuclear factor κB (NF-κB) and contributes to PD-L1 up-regulation in tumor-associated macrophages. (B) *S*-palmitoylation of interferon-γ (IFN-γ) receptor 1 (IFNGR1) at Cys122 triggers adaptor-related protein complex 3 subunit delta 1 (AP3D1)-dependent lysosomal sorting, disrupting IFN-γ/Janus kinase 1 (JAK1)/signal transducer and activator of transcription 1 (STAT1) signaling and reducing major histocompatibility complex class I (MHC-I) expression. Fatty acid synthase (FASN)-driven palmitoylation (Palm) accelerates MHC-I degradation, whereas inhibition of FASN or palmitoylation restores antigen presentation and enhances CD8^+^ T cell cytotoxicity. (C) Left: Double-stranded DNA (dsDNA) activates cyclic GMP–AMP (cGAMP) synthase (cGAS) (Cys404/405) to generate cGAMP from adenosine triphosphate (ATP)/guanosine triphosphate (GTP), which activates stimulator of interferon genes (STING) and triggers the interferon signaling pathway. Depalmitoylation of cGAS mediated by lysophospholipase-like 1 (LYPLAL1) inhibits cGAMP production and suppresses downstream interferon signaling pathway activation. Right: Mitochondrial antiviral signaling protein (MAVS) (Cys15/Cys465) is regulated by palmitoylation status. Nonpalmitoylation of MAVS fails to activate the interferon signaling pathway, whereas *S*-palmitoylation promotes mitochondrial membrane surface and oligomerization of MAVS, enabling activation of the interferon signaling pathway. (D) *S*-palmitoylation of vacuole membrane protein 1 (VMP1) promotes small extracellular vesicle (sEV) biogenesis and export of immunosuppressive signals. Palmitoylated interferon-induced transmembrane protein 2 (IFITM2) is selectively enriched in sEVs, which inhibit IFN responses in dendritic cells. Numbers indicate intervention points: (1) inhibition of IFITM2 *S*-palmitoylation (palmitoylation inhibitor), reducing its loading into exosomes, and (2) inhibition of exosome biogenesis/release (prevent release), blocking transfer of palmitoylated IFITM2 to dendritic cells. PD-L1 is also packaged into sEVs in a palmitoylation-dependent manner, suppressing CD8^+^ T cell activity and promoting regulatory T cell (Treg) expansion. Arrows indicate activation or positive regulation, whereas the inhibition symbol (⊣) indicates suppression/negative regulation. Illustration was created using BioRender.com. MVB, multivesicular bodies; PD-1, programmed cell death protein 1; STIM-3, stromal interaction molecule 3; TLRs, Toll-like receptors.

PD-L1 is *S*-palmitoylated at Cys272 in its cytoplasmic tail, a modification essential for its retention on the cancer cell surface (Fig. 5A). Yao et al. [15] demonstrated that DHHC3 and DHHC9 predominantly catalyze this modification, thereby inhibiting PD-L1 endocytosis and lysosomal degradation, prolonging its half-life, and enhancing its immunoinhibitory function. In various cancer models, high DHHC3 expression levels are correlated with elevated PD-L1 membrane levels. Disruption of *S*-palmitoylation via DHHC3 knockout or treatment with the palmitoylation inhibitor 2-bromopalmitate (2-BP) promotes PD-L1 ubiquitination and degradation, thereby restoring CD8^+^ T cell activation and cytotoxicity [15]. Furthermore, Wang et al. [150] identified DHHC3 as the key acyltransferase responsible for PD-L1 *S*-palmitoylation and discovered a marine-derived natural compound, benzosceptrin C (BC), which inhibits its enzymatic activity.

Palmitoylated PD-L1 shows enhanced affinity for the membrane-stabilizing factor chemokine-like factor (CKLF)-like MAL and related proteins for vesicle trafficking and membrane link (MARVEL) transmembrane-domain-containing protein 6 (CMTM6), which interferes with E3-ubiquitin-ligase-mediated polyubiquitination and prevents PD-L1 trafficking to lysosomes [151]. Therefore, CMTM6 acts as a functional chaperone that synergizes with palmitoylated PD-L1 to maintain high surface expression, thereby enabling sustained programmed cell death protein 1 (PD-1) engagement and suppression of T cell effector function (Fig. 5A).

In addition to regulating PD-L1, *S*-palmitoylation also regulates other immune checkpoint molecules. For instance, *S*-palmitoylation of T cell immunoglobulin and mucin-domain-containing protein 3 (TIM3) enhances its membrane localization on dendritic cells and impairs antigen presentation, thereby inhibiting T cell priming (Fig. 5A) [151]. CD47, a key “don’t-eat-me” signal, contains predicted palmitoylation sites from the SwissPalm database (https://swisspalm.org/), which may influence its interaction with signal regulatory protein α and macrophage-mediated phagocytosis [151]; however, this requires further experimental validation. Notably, myeloid differentiation primary response 88 (MYD88), best known for its role in proinflammatory signaling, is also implicated in immune checkpoint regulation. Activation of the Toll-like receptor (TLR)/nuclear factor κB (NF-κB) pathway via MYD88 up-regulates PD-L1 expression, contributing to the immunosuppressive phenotype of tumor-associated macrophages [152,153]. Because MYD88 activity is modulated by *S*-palmitoylation [154], it may indirectly reinforce PD-L1 expression, thereby amplifying immune evasion in a feedforward manner. Mechanistically, downstream of TLRs, MYD88 is also regulated by *S*-palmitoylation: DHHC6-mediated *S*-palmitoylation at Cys113 and Cys274 facilitates MYD88 membrane recruitment and interaction with interleukin-1 (IL-1) receptor-associated kinase 4 (IRAK4), thereby sustaining NF-κB and mitogen-activated protein kinase activation and proinflammatory cytokine expression [154]. Together, these events establish an *S*-palmitoylation-dependent TLR9–MYD88 module that may reinforce chronic inflammatory signaling and contribute to an immunosuppressive niche (Fig. 5A).

#### Regulation of antigen presentation function (adaptive immunity)

Effective CD8^+^ T cell activation relies on the integrity of the major histocompatibility complex class I (MHC-I) antigen-processing and presentation pathways, which govern peptide loading and surface display [155–157]. Recent studies have demonstrated that *S*-palmitoylation directly regulates this pathway in tumor cells. For example, DHHC3-mediated *S*-palmitoylation promotes the lysosomal degradation of MHC-I heavy chains, thereby reducing the stability of surface MHC-I and impairing antigen presentation [158]. Altered lipid palmitate metabolism also diminishes peptide availability and limits the diversity of presented antigens. In antigen-presenting cells, such as dendritic cells and macrophages, *S*-palmitoylation may additionally modulate vesicular trafficking, receptor signaling pathways, and endosomal handling of peptide–MHC-I complexes, which influence antigen-presenting cell maturation and cross-presentation efficiency. Such *S*-palmitoylation-driven defects weaken the priming of CD8^+^ T cells, lowering T cell receptor signaling strength, restraining clonal expansion, and ultimately promoting immune evasion.

Interferon-γ (IFN-γ) initiates this pathway by activating Janus kinase 1 (JAK1)/STAT1 signaling through its receptor, IFN-γ receptor 1 (IFNGR1), resulting in MHC-I up-regulation. *S*-palmitoylation of IFNGR1 at Cys122 enhances its interaction with adaptor-related protein complex 3 subunit delta 1 (AP3D1), promoting receptor endocytosis and lysosomal degradation, thereby attenuating downstream signaling and reducing MHC-I expression (Fig. 5B) [159]. These findings highlight the pivotal role of *S*-palmitoylation in IFNGR1 stability–IFN-γ signaling–MHC-I regulation axis. The adaptor protein optineurin competes with AP3D1 for IFNGR1 binding, thereby protecting the receptor from degradation and sustaining pathway activity. Conversely, the loss of optineurin accelerates IFNGR1 degradation and diminishes antigen presentation capacity [159].

FASN, the primary enzyme generating palmitoyl–CoA, indirectly contributes to MHC-I regulation (Fig. 5B). In hepatocellular carcinoma models, FASN-driven palmitoylation promotes MHC-I degradation via lysosomal pathways, whereas pharmacological inhibition of FASN (e.g., orlistat and TVB-2640) stabilizes MHC-I, enhances CD8^+^ T cell cytotoxicity, and synergizes with anti-PD-L1 therapy (Fig. 5B) [158]. In colorectal cancer, treatment with the pan-palmitoylation inhibitor 2-BP restores MHC-I expression and reactivates IFN-γ signaling, reinforcing the multifaceted role of *S*-palmitoylation in shaping antigen presentation [160].

#### PRR signaling and innate immune sensing in the TME

Innate immune signaling serves as the first line of defense against tumors and pathogens. In the TME, PRRs, such as TLRs, cyclic guanosine monophosphate (GMP)–AMP (cGAMP) synthase (cGAS), stimulator of IFN genes (STING), and mitochondrial antiviral signaling protein (MAVS), orchestrate type I IFN (IFN-I) production and inflammatory responses in both tumor and tumor-associated myeloid cells [161–163]. Their activation is driven by tumor-derived damage-associated molecular patterns (DAMPs) and, in some contexts, microbial pathogen-associated molecular patterns, which determine whether the TME becomes immunostimulatory or shifts toward chronic immunosuppressive inflammation. *S*-palmitoylation is a key modulator of these pathways by regulating protein activation, membrane localization, and signaling efficiency, thereby weakening early immune surveillance and facilitating tumor immune evasion.

The cGAS–STING axis is the principal cytosolic DNA-sensing pathway that induces IFN-I expression. cGAS is *S*-palmitoylated at conserved cysteines (Cys404 and Cys405) by DHHC9, which promotes dimerization, enhances double-stranded DNA binding, and boosts cGAMP synthesis (Fig. 5C) [17]. The APT lysophospholipase-like 1 (LYPLAL1) acts as a cognate depalmitoylase and removes this lipid modification, thereby inhibiting cGAS enzymatic activity and IFN-I output [17]. Genetic or pharmacological inhibition of LYPLAL1 sustains cGAS *S*-palmitoylation, promotes intratumoral IFN-I responses, increases PD-L1 expression, and improves the efficacy of PD-1 blockade in vivo [17]. These data reveal that dynamic cGAS palmitoylation–depalmitoylation functions as a druggable checkpoint in innate immune regulation.

In addition, STING activation depends on *S*-palmitoylation at Cys91, which drives its oligomerization on the ER membrane and activation of TRAF family member-associated NF-κB activator (TANK)-binding kinase 1 and IFN regulatory factor 3 downstream. The metabolite 4-octyl itaconate covalently modifies Cys91, blocks STING *S*-palmitoylation, and inhibits signaling [164]. In RNA virus-induced pathways, MAVS *S*-palmitoylation at Cys15 and Cys465 by DHHC5 and DHHC16 anchors the adaptor to the mitochondrial outer membrane and enables its oligomerization, whereas the loss of this modification impairs IFN-I responses (Fig. 5C) [165]. Collectively, *S*-palmitoylation fine-tunes the activation thresholds, subcellular localization, and signal duration of cGAS, STING, and MAVS within the TME.

#### Regulation of intercellular communication and exosomes

Intercellular communication, particularly via exosome-mediated signaling, plays a crucial role in establishing and sustaining an immunosuppressive TME [166–168]. In this context, *S*-palmitoylation is a key modulator influencing the formation, secretion, and cargo sorting of tumor-derived exosomes that propagate immune evasion signals across cellular compartments.

Vesicle membrane protein 1 (VMP1) is a central regulator of small extracellular vesicle (sEV) biogenesis. *S*-palmitoylation at Cys263 and Cys278, mediated by DHHC3, promotes the interaction of VMP1 with apoptosis-linked gene 2-interacting protein X, a critical component of the endosomal sorting complex required for transport, thereby facilitating multivesicular body maturation and efficient exosome release (Fig. 5D) [169]. Disruption of this modification impairs exosome secretion and limits their role in modulating the tumor immune microenvironment.

IFN-induced transmembrane protein 2 (IFITM2) is another exosome-enriched protein whose *S*-palmitoylation enhances its packaging into vesicles [170]. When transferred to dendritic cells via tumor-derived exosomes, palmitoylated IFITM2 suppresses IFN-α signaling, thereby inhibiting innate antiviral and antitumor responses [170]. However, inhibition of IFITM2 *S*-palmitoylation or exosome biogenesis reverses this suppression and restores immune vigilance (Fig. 5D).

*S*-palmitoylation also enhances the exosomal transport of immune checkpoint proteins. After *S*-palmitoylation at Cys272, PD-L1 is stabilized on the tumor cell membrane and preferentially sorted into exosomes, where it contributes to long-range immunosuppressive signaling. These PD-L1-enriched exosomes inhibit cytotoxic T cell function at distant sites and promote regulatory T cells (Tregs) expansion, amplifying systemic immune escape (Fig. 5D) [15].

Collectively, these examples illustrate the mechanisms by which palmitoylation-dependent control of VMP1, IFITM2, and PD-L1 organizes exosome biogenesis and cargo loading to propagate immunosuppressive signals across the TME (Fig. 5D).

Overall, *S*-palmitoylation exerts widespread and coordinated control over the tumor immune microenvironment. At the adaptive level, it stabilizes immune checkpoint molecules such as PD-L1, TIM3, and CD47 and accelerates degradation of IFN-γ receptors and MHC-I, thereby weakening antigen presentation and CD8^+^ T-cell-mediated recognition. At the innate level, *S*-palmitoylation governs the activity of core sensors, including cGAS, STING, MAVS, and TLR9–MYD88, shaping the magnitude and persistence of IFN-Is and inflammatory outputs. Simultaneously, *S*-palmitoylation of VMP1, IFITM2, and PD-L1 organizes exosome biogenesis and cargo sorting, facilitating long-range delivery of inhibitory ligands and reinforcing an immunosuppressive niche. These processes together establish an immune escape feedback loop characterized by “membrane stabilization–signal modulation–intercellular propagation”.

### Regulatory role of protein *S*-palmitoylation in cancer progression within the inflammatory microenvironment

**Fig. 6.**
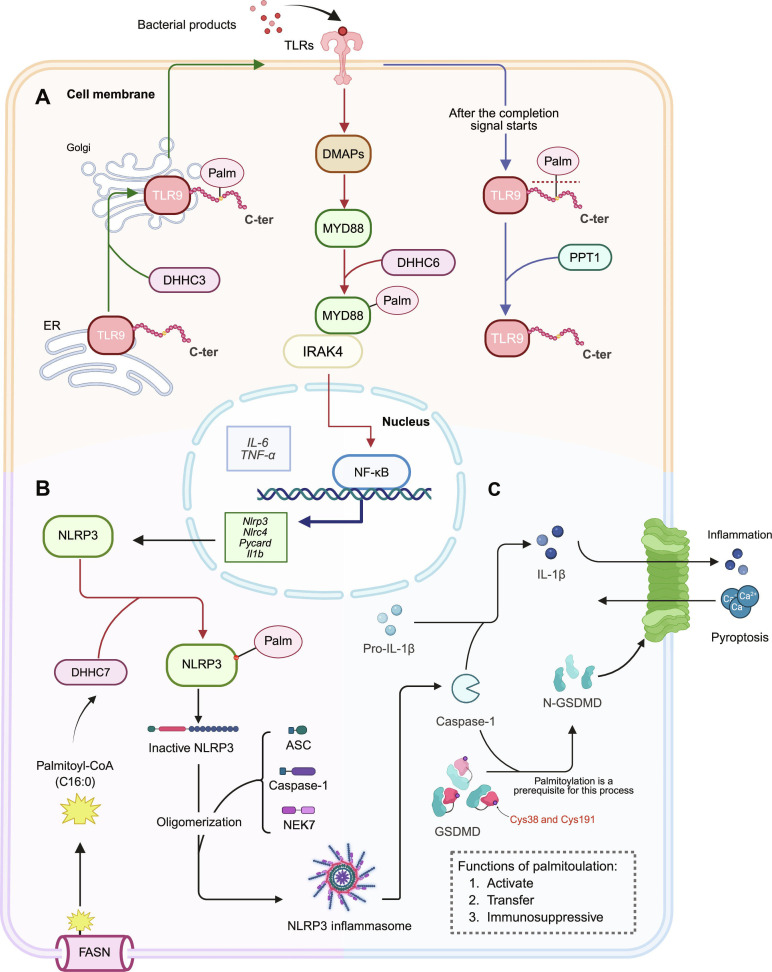
*S*-palmitoylation fine-tunes the Toll-like receptor (TLR)–NOD-like receptor family pyrin-domain-containing protein 3 (NLRP3)–gasdermin D (GSDMD) inflammatory signaling axis under the tumor inflammatory microenvironment. (A) Upon microbial stimulation, TLR9 is palmitoylated at its C-terminal domain by DHHC3, promoting endoplasmic reticulum (ER)-to-Golgi trafficking and membrane anchoring. Following signal transduction, depalmitoylation by palmitoyl-protein thioesterase 1 (PPT1) facilitates activation of myeloid differentiation primary response 88 (MYD88). MYD88 is palmitoylated at cysteine-113 (Cys113) and Cys274 by DHHC6, enhancing its membrane localization and interaction with interleukin-1 (IL-1) receptor-associated kinase 4 (IRAK4), thereby activating nuclear factor κB (NF-κB) signaling and inducing IL-6 and tumor necrosis factor-α (TNF-α) transcription. (B) *S*-palmitoylation of NLRP3 at Cys126 and Cys898, catalyzed by DHHC7 and dependent on palmitoyl–CoA synthesized by fatty acid synthase (FASN), facilitates its translocation to the Golgi and assembly of the inflammasome complex with apoptosis-associated speck-like protein containing a CARD (ASC), cysteine-aspartic acid protease 1 (caspase-1), and never in mitosis gene A (NIMA)-related kinase 7 (NEK7), promoting IL-1β maturation and amplifying inflammatory responses. C16:0 denotes a 16-carbon saturated fatty acyl chain (0 double bonds), i.e., palmitate/palmitoyl. (C) Following cleavage by caspase-1, GSDMD requires *S*-palmitoylation at Cys38 and Cys191 for membrane localization and pore formation, which is essential for executing pyroptosis and establishing an immunosuppressive inflammatory microenvironment. Illustration was created using BioRender.com. Ca^2+^, calcium ion; C-ter, C terminus; DMAPs, 4-dimethylaminopyridines; Il1b, interleukin-1β; N-GSDMD, N-terminal gasdermin D; Nlrc4, NOD-like receptor family CARD domain containing 4; Palm, photoactivated localization microscopy; pro-IL-1β, pro-IL-1β; Pycard, PYD and CARD domain containing.

The inflammatory microenvironment plays a key role in cancer initiation, progression, and immune evasion. *S*-palmitoylation extensively regulates major components of inflammatory signaling pathways, particularly by amplifying signals through TLRs, nucleotide-binding oligomerization domain (NOD)-like receptor family pyrin-domain-containing protein 3 (NLRP3) inflammasomes, and gasdermin D (GSDMD) [66,154,171,172]. Notably, the TLR9–MYD88 module described above is integrated into this network (schematically summarized in Fig. 6A) [66,154]. These 3 components constitute a progressive cascade of proinflammatory activation, with *S*-palmitoylation spanning the stages of signal recognition, amplification, and execution, profoundly influencing immune remodeling within the TME.

#### *S*-palmitoylation-mediated regulation of the TLR signaling pathway

TLRs are key innate immune receptors that recognize pathogen-associated molecular patterns and DAMPs, activate inflammatory cascades such as NF-κB, and promote the release of proinflammatory cytokines. Within the inflammatory TME, aberrant activation of TLR signaling enhances cancer cell proliferation, immunosuppressive niche formation, and maintenance of CSC properties [173].

*S*-palmitoylation can strengthen TLR-driven inflammatory signaling by modulating the localization and activation competency of pathway components. Ni et al. [66] demonstrated that TLR9 undergoes dynamic *S*-palmitoylation at the C-terminal region of its transmembrane domain, catalyzed by DHHC3, which promotes ER-to-Golgi trafficking and membrane anchoring to support efficient ligand sensing (Fig. 6A). Upon pathway activation, PPT1-mediated depalmitoylation removes this modification, enabling exposure/engagement of the intracellular signaling domain and triggering downstream NF-κB activation with tumor necrosis factor-α (TNF-α) and IL-6 production (Fig. 6A) [66]. In parallel, the downstream adaptor MYD88 is also regulated by *S*-palmitoylation. Leishman et al. [154] reported that DHHC6 catalyzes MYD88 *S*-palmitoylation at Cys113 and Cys274, facilitating membrane localization and interaction with IRAK4, thereby promoting NF-κB and mitogen-activated protein kinase activation and sustaining IL-6 and TNF-α expression (Fig. 6A). Together, these findings indicate that *S*-palmitoylation is critical for the intensity and persistence of TLR signaling and provides a proinflammatory foundation for subsequent NLRP3 inflammasome activation.

#### *S*-palmitoylation-mediated regulation of NLRP3 inflammasome

The NLRP3 inflammasome functions as an amplification hub for inflammatory signaling and plays a critical role in cancer-associated inflammation. Upon activation, it promotes caspase-1-mediated cleavage of pro-IL-1β and pro-IL-18, releasing large amounts of inflammatory cytokines that drive immune cell recruitment, inflammatory persistence, and an immunosuppressive microenvironment [174,175].

NLRP3 activity is regulated by multisite *S*-palmitoylation. Yu et al. [172] showed that DHHC7 palmitoylates NLRP3 at Cys126, enhancing its anchoring to the trans-Golgi network and trafficking to dispersed trans-Golgi network, thereby facilitating apoptosis-associated speck-like protein containing a caspase recruitment domain (ASC) binding and rapid inflammasome assembly (Fig. 6B). Leishman et al. [154] further demonstrated that *S*-palmitoylation at Cys898 contributes to NLRP3 structural stability and stimulus responsiveness, representing another critical regulatory site.

*S*-palmitoylation of NLRP3 is closely associated with lipid metabolism. FASN is frequently overexpressed in many cancers and drives de novo palmitate synthesis, which can be converted to palmitoyl–CoA by long-chain acyl–CoA synthetases, thereby supplying a major intracellular pool of palmitoyl–CoA [176,177]. Elevated levels of FASN enhance NLRP3 *S*-palmitoylation, promote inflammasome assembly and IL-1β secretion, and are associated with exacerbated inflammatory states in tumor models (Fig. 6B and C) [154]. Activated inflammasomes support cancer progression by releasing inflammatory cytokines, recruiting immunosuppressive cells, such as myeloid-derived suppressor cells, Tregs, M2-polarized macrophages, and activating survival-promoting pathways, such as the STAT3 pathway [178,179]. Although the specific mechanisms remain unclear, several studies have implicated NLRP3 in metastasis, angiogenesis, and immunotherapy resistance [180–183].

#### Protein *S*-palmitoylation of GSDMD—The executioner of pyroptosis

GSDMD is a downstream effector of the NLRP3 inflammasome signaling. Following cleavage by caspase-1, its N-terminal fragment forms membrane pores, triggering pyroptosis and release of IL-1β, IL-18, and DAMPs, which further amplify local inflammation (Fig. 6C) [184,185].

Balasubramanian et al. [171] reported that GSDMD undergoes *S*-palmitoylation at Cys191 and Cys38, which enhances membrane binding and pore formation by the cleaved N-terminal domain, an essential step for pyroptotic activity. FASN supplies palmitoyl–CoA, which is necessary for this modification [154]. In addition, Du et al. [186] discovered that GSDMD undergoes reactive-oxygen-species-dependent *S*-palmitoylation under oxidative stress, affecting both the cleaved and full-length forms and enhancing their stability and membrane localization efficiency.

GSDMD pyroptosis not only releases proinflammatory factors but is also associated with an immunosuppressive state. In mouse tumor models, enhanced palmitoylation-mediated GSDMD activity is closely associated with myeloid-derived suppressor cell enrichment and reduced CD8^+^ T cell infiltration [171,187]. This suggests that GSDMD is involved in immune evasion. However, evidence is currently lacking for its direct regulation of tumor cell proliferation and stemness maintenance, and its indirect effects warrant further investigation. In summary, regulation of GSDMD by *S*-palmitoylation establishes essential connections among lipid metabolism, inflammatory signaling, and tumor immune regulation.

## Cross-talk between Protein *S*-Palmitoylation and Other PTMs in Cancer

Protein *S*-palmitoylation operates within a broader PTM network rather than as an isolated modification. In cancer-stemness-related pathways such as Wnt/β-catenin, Hippo, TGF-β, and Hh pathways, core receptors and transcriptional regulators are simultaneously controlled by phosphorylation, ubiquitination, acetylation, and glycosylation, in addition to *S*-palmitoylation [9,188]. Recent PTM-centered reviews in cancer signaling and immunity highlight that these modifications rarely act alone but form combinatorial “codes” that collaboratively tune protein activity, stability and signaling output in malignant cells and TME [9,189]. In this context, reversible *S*-palmitoylation provides a lipid anchor that repositions proteins within specific membrane microdomains, thereby altering their access to kinases, phosphatases, and E3 ubiquitin ligases and indirectly reshaping phosphorylation and ubiquitination patterns on CSC-related effectors [190,191]. Mechanistically, *S*-palmitoylation can stabilize membrane-associated receptors or scaffold proteins and shield degradation motifs, delaying ubiquitin-mediated turnover, whereas loss of *S*-palmitoylation may expose degrons and facilitate proteasomal or lysosomal degradation [9]. For transcriptional regulators, *S*-palmitoylation-driven changes in subcellular localization can cooperate with acetylation-dependent control of chromatin association to fine-tune transcriptional programmes that sustain stemness and therapy resistance [9]. Similar PTM cross-talk has been reported for immune checkpoint molecules and innate immune sensors, where palmitoylation intersects with phosphorylation, glycosylation, and ubiquitination to control protein trafficking and signaling competence [192–194]. Together, these observations support the view that protein *S*-palmitoylation shapes cancer stemness not only through direct effects on individual substrates but also by integrating into multilayered PTM networks that coordinate CSC maintenance, plasticity, and resistance to therapy. The principal regulatory levels at which *S*-palmitoylation interacts with other PTMs in cancer are summarized in Table 3.

**Table 3.** Cross-talk between protein *S*-palmitoylation and other posttranslational modifications in cancer

Biological level/process	Role of protein *S*-palmitoylation	Partner PTMs typically involved	Canonical functional consequence in cancer, CSCs, and TME	Representative pathways or proteins
Receptor/co-receptor signaling at the plasma membrane	Provides reversible lipid anchor, stabilizes receptors in specific membrane microdomains, promotes ligand binding and signal initiation	Phosphorylation of cytoplasmic tails; ubiquitination controlling endocytosis and degradation; N-glycosylation affecting folding and ligand affinity	Fine-tuned control of receptor abundance and signaling amplitude; sustained activation favors proliferation, EMT and CSC maintenance	Wnt–Frizzled–LRP5/6, TGF-β/BMP receptors, PD-1/PD-L1 axis
Adaptor and scaffold proteins in signalosomes	Supports assembly of multiprotein complexes by increasing local concentration at membranes or organelles	Phosphorylation-dependent recruitment of SH2/SH3-containing adaptors; ubiquitination shaping complex turnover	Stabilization of oncogenic signalosomes and inflammasomes, prolonged downstream kinase activation, and inflammatory output	MYD88, components of the TLR/NF-κB and NLRP3 inflammasome pathways
Transcriptional coregulators and nuclear effectors	Modulates protein conformation and interaction interfaces, indirectly impacting transcriptional complex formation and chromatin engagement	Phosphorylation controlling nuclear-cytoplasmic shuttling; acetylation on histones and transcription factors; ubiquitination-mediated turnover	Reprogramming of transcriptional networks that sustain stemness, metabolic plasticity and therapy resistance	TEAD–YAP/TAZ complex in Hippo signaling pathway; SMAD proteins in TGF-β/BMP pathway; *GLI* factors in Hh pathway
Metabolic enzymes and stress-response mediators	Repositions metabolic enzymes to membranes or vesicles, facilitating metabolic rewiring and adaptation to stress	Phosphorylation controlling enzyme activity; ubiquitination governing degradation; acetylation regulating metabolic enzyme function	Support of bioenergetic demands of CSCs and adaptation to hypoxia, nutrient deprivation, or oxidative stress	HK1 and other glycolytic/mitochondrial enzymes; FASN and lipid metabolic regulators
Immune checkpoint regulation at the tumor-immune interface	Stabilizes immune checkpoint proteins at the cell surface and limits their lysosomal or proteasomal degradation	N-glycosylation modulating ligand-receptor affinity; phosphorylation creating docking sites for E3 ligases; ubiquitination directing internalization and degradation	Maintenance of high checkpoint expression, suppression of T cell activity, and impaired response to immunotherapy	PD-L1/PD-1 and other coinhibitory receptors
Innate immune sensing and inflammatory signaling	Defines membrane platforms and activation thresholds for nucleic acid sensors and adaptor proteins.	Phosphorylation and K48/K63-linked ubiquitination controlling activation and signal termination; SUMOylation/deSUMOylation in some contexts.	Balanced control between antitumor IFN responses and chronic, immunosuppressive inflammation in the TME	cGAS–STING axis; TLR/NF-κB and TLR/NLRP3 cascades

BMP, bone morphogenetic protein; cGAS, cyclic GMP–AMP synthase; CSCs, cancer stem cells; EMT, epithelial–mesenchymal transition; FASN, fatty acid synthase; *GLI*, *GLI* family zinc finger transcription factors; HK1, hexokinase 1; IFN, interferon; LRP5/6, low-density lipoprotein receptor-related protein 5/6; MYD88, myeloid differentiation primary response 88; NF-κB, nuclear factor κB; NLRP3, NOD-like receptor family pyrin-domain-containing protein 3; PD-1, programmed cell death protein 1; PD-L1, programmed death-ligand 1; SMAD, SMA- and MAD-related protein proteins; STING, stimulator of interferon genes; TAZ, transcriptional coactivator with PDZ-binding motif; TEAD, transcriptional enhanced associate domain-containing transcription factor; TGF-β, transforming growth factor-β; TLR, Toll-like receptor; TME, tumor microenvironment; Wnt, Wingless/Integrated; YAP, Yes-associated protein.

## Challenges in Therapeutic Targeting of Protein *S*-Palmitoylation

### Inhibition of *S*-palmitoylation-related enzymes

Compared to other lipidation enzymes, palmitoyl transferases are notably more numerous, and our current understanding of potential redundancy among DHHC-PAT family members remains limited. This complexity hampers the development of selective inhibitors and underscores the urgent need to better characterize these enzymes. To date, only a few DHHC proteins have had their crystal structures resolved, and this process is essential to guide rational drug design. Despite the fact that the DHHC domain contains a conserved zinc finger motif, few inhibitors with specificity for individual DHHCs have been identified, and none have received clinical approval. Currently, most DHHC inhibitors used in preclinical models, such as 2-BP, lack specificity and act broadly across the enzyme family. While targeted gene knockouts and chemical inhibition strategies have shown utility in animal models, the pharmacological control of specific DHHCs remains elusive. Whether DHHC enzymes can be exploited as viable therapeutic targets in human diseases is an open question.

Table 4 summarizes a list of compounds modulating *S*-palmitoylation in preclinical research. Despite promising activity, most have not progressed beyond early-stage investigation. In dynamic *S*-palmitoylation studies, the APT inhibitor palmostatin B is widely used to inhibit depalmitoylation and track *S*-palmitoylation turnover (Table 5). However, 2-BP exhibits substantial cytotoxicity, a limitation that has long been recognized but often overlooked in practice. Careful titration remains essential when using 2-BP in cellular systems [117].

**Table 4.** Palmitoyl transferase inhibitor

Inhibitor	Mechanism	Effects or applications	Research progress stage ^a^	Ref.
Compound V	Inhibits DHHC2 and DHHC9 autoacylation	Compared with 2-BP, it was reversible	In vitro	[225]
2-BP	Irreversibly inhibits DHHCs	Inhibits tumor cell proliferation and tumor growth	Mechanistic tool	[15]
PD-PALM peptide	Competitive peptide derived from PD-L1 palmitoylation motif	Promotes PD-L1 degradation and enhances T cell killing	Preclinical (in vivo)	[15]
Cyano-myracrylamide	Engages with DHHCs and inhibits protein *S*-palmitoylation	Inhibits tumor cell proliferation and tumor growth and has decreased toxicity than 2-BP	Preclinical (in vivo)	[195]
Prilocaine	Weakens DHHC15 transcripts	Inhibits tumor cell proliferation, self-renewal, and tumor growth	In vitro	[226]
Lidocaine	Weakens DHHC15 transcripts	Inhibits tumor cell proliferation, self-renewal, and tumor growth	In vitro	[226]
Procaine	Weakens DHHC15 transcripts	Inhibits tumor cell proliferation, self-renewal, and tumor growth	In vitro	[226]
Ropivacaine	Weakens DHHC15 transcripts	Inhibits tumor cell proliferation, self-renewal, and tumor growth	In vitro	[226]
Artemisinin	Covalently binds and inhibits DHHC6	Reduces the palmitoylation of the oncogenic protein NRAS and anticancer	Preclinical (in vivo)	[196]
Lanatoside C	Increased stability of DHHC21	FDA-approved small-molecule compounds targeting DHHC enzymes	Clinical (repurposed)	[87]
Benzosceptrin C	Inhibiting DHHC3 enzymatic activity	Prevents palmitoylation of PD-L1 by inhibiting DHHC3 enzymatic activity	Preclinical (in vivo)	[150]

^a^ In vitro refers to cell-culture-based assays performed outside a living organism; mechanistic tool refers to research-use pharmacological tool.

FDA, Food and Drug Administration; NRAS, neuroblastoma RAS viral oncogene homolog; PD-L1, programmed death-ligand 1; DHHC, Asp–His–His–Cys motif; 2-BP, 2-bromopalmitate.

**Table 5.** Depalmitoylase inhibitors

Inhibitors	Mechanisms	Effects or applications	Research progress stages^a^	Refs.
Palmostatin B	Broad-spectrum inhibitor of APT1/2, PPT1, ABHD17A/B/C, and others	Selectively influencing RAS gene	Mechanistic tool	[21,227,228]
Palmostatin M	Broad-spectrum inhibitor of APT1/2, PPT1, ABHD17A/B/C, and others	Broad-spectrum depalmitoylase inhibitor	Mechanistic tool	[21,227,228]
ML211	Inhibition of APT1, APT2, ABHD11, and PPT1	Influence on depalmitoylase activity	Preclinical (in vivo)	[67]
ML348	Inhibition of APT1	Restores defective huntingtin trafficking; affects cardiac infarct	Preclinical (in vivo)	[229,230]
ML349	Inhibition of APT2	Represses UVB-induced melanomagenesis	Preclinical (in vivo)	[231]
ABD957	Inhibition of ABHD17s	Inhibits NRas-mutant AML cell growth	Preclinical (in vivo)	[21]
HCQ	Inhibition of PPT1	Enhances AEG-1 palmitoylation and degradation and inhibits HCC	Preclinical (in vivo)	[86]
GNS561	Inhibition of PPT1	Specifically inhibits PPT1 and induces cancer cell death	Preclinical (in vivo)	[232]
Dimeric chloroquine 661 (DC661)	Inhibition of PPT1	Inhibits PPT1 and enhances cancer survival	Preclinical (in vivo)	[72]
Dimeric quinacrines 661 (DQ661)	Inhibition of PPT1	Blocks mTOR and autophagy in unique way	Preclinical (in vivo)	[233]
LYPLAL1 inhibitor	Blocks cGAS depalmitoylation	Enhances antitumor immunity	Preclinical (in vivo)	[17]

^a^ Mechanistic tool refers to a research-use pharmacological tool.

ABHD11, α/β hydrolase domain containing 11; AML, acute myeloid leukemia; APT1/2, acyl–protein thioesterase 1/2; cGAS, cyclic GMP–AMP synthase; mTOR, mammalian target of rapamycin; NRas-mutant, neuroblastoma RAS viral oncogene homolog (mutant); PPT1, palmitoyl-protein thioesterase 1; RAS, rat sarcoma viral oncogene homolog; UVB, ultraviolet B.

To overcome such drawbacks, Yao et al. [15] developed a competitive peptide derived from the PD-L1 *S*-palmitoylation motif, which substantially reduced PD-L1 expression. In addition, cyano-myracrylamide, a recently described broad-spectrum depalmitoylation inhibitor, has been optimized to mitigate 2-BP-associated toxicity and nonspecific effects on APTs [195]. Drugs from unrelated therapeutic areas have also been found to interfere with *S*-palmitoylation. For instance, the antimalarial agent artemisinin binds covalently to DHHC6 and disrupts its catalytic function, reducing NRas *S*-palmitoylation and potentially suppressing downstream oncogenic signaling [196]. A cardiac glycoside, lanatoside C, was recently shown to stabilize DHHC21, restoring its tumor-suppressive activity in diffuse large B cell lymphoma [87]. Beyond DHHCs, recent work in melanoma has revealed that acute pharmacologic inhibition of the lysosomal depalmitoylase PPT1 potently blocks lysosomal function and tumor growth, whereas chronic inhibition induces adaptive responses that attenuate antitumor efficacy [197]. This divergence highlights the strong time and context dependence of targeting depalmitoylases in vivo.

The Hippo–YAP pathway, frequently dysregulated in YAP-driven cancers such as mesothelioma, has remained notoriously difficult to target. As key downstream effectors, YAP and TAZ promote prosurvival gene expression by binding TEAD transcription factors in the nucleus. The discovery that TEADs require self-palmitoylation for activity has spurred the development of pan-TEAD palmitoylation inhibitors. These molecules block TEAD *S*-palmitoylation, disrupting the YAP/TAZ-TEAD complex and suppressing oncogenic transcription. In 2021, Tang et al. [107] identified a small molecule that selectively inhibits TEAD *S*-palmitoylation, thereby blocking YAP/TAZ-TEAD interactions and downstream gene expression. More recently, GNE-7883 was reported as an allosteric TEAD inhibitor that binds the *S*-palmitoylation site and prevents YAP/TAZ binding across all TEAD paralogs [198]. This compound reduces chromatin accessibility at TEAD-binding motifs, inhibits proliferation across multiple cell lines, and exhibits potent antitumor efficacy in vivo. Advances in this area are summarized in Table 6.

**Table 6.** Other types of inhibitors

Inhibitors	Mechanisms	Effects or applications	Research progress Stages ^a^	Target substrates	Ref.
LGK974	PORCN inhibitor Inhibition of Wnt ligand palmitoylation	Targets Wnt-driven cancers through PORCN inhibition	Mechanistic tool	Transferase	[234]
Cerulenin	FASN inhibitor	Potentiates TKIs’ proapoptotic effects	Preclinical (in vivo)	PORCN	[235]
H-151	Occupies palmitoylation site on STING	Inhibits STING palmitoylation for autoimmune disease	Preclinical (in vivo)	FASN	[236]
Compound2	Binds TEAD lipid pocket and disrupts *S*-palmitoylation	Impacts TEAD stability in Hippo pathway cancers	Preclinical (in vivo)	STING	[237]
Metformin	Inhibits FASN-dependent palmitoylation	Potential against inflammation via palmitoylation regulation	Preclinical (in vitro)	TEAD	[238]
JM17	Inhibits TEAD palmitoylation and destabilizes TEAD	Impairs proliferation and migration in YAP-active cancers	Clinical (repurposed)	FASN	[239]
MGH-CP1	Inhibits TEAD palmitoylation	Blocks cancer stemness, organ growth, and tumor initiation	Preclinical (in vitro)	TEAD	[108]
TM2	Potent inhibition of TEAD autopalmitoylation	Antiproliferation in YAP-dependent cancer cells	Preclinical (in vivo)	TEAD	[240]
PCSK9-derived peptide	Competitively inhibits PCSK9 palmitoylation	Suppresses AKT and enhances sorafenib effect in HCC	Preclinical (in vitro)	TEAD	[207]
GNE-7883	Allosterically blocks YAP/TAZ-TEAD via TEAD lipid pocket	Suppresses proliferation in vitro, strong antitumor efficacy in vivo	Preclinical (in vivo)	Substrate peptide	[198]
BI-2536	Modulates palmitoylation-related genes/enzymes	Modulates palmitoylation for cancer treatment	Preclinical (in vivo)	TEAD	[241]
Etoposide, piperlongumine	Modulates palmitoylation-related genes/enzymes	Modulates palmitoylation for cancer treatment	Clinical (repurposed)	Indirect modulator	[241]
4-Octyl itaconate	Blocks STING palmitoylation	Inhibits autoimmune activation	Preclinical (in vitro)	Indirect modulator	[164]
Orlistat	Restricts palmitic acid synthesis	Antagonizes liver tumorigenesis via AKT depalmitoylation	Preclinical (in vivo)	STING	[58]
YTHDF3-derived peptide	Competitively inhibits YTHDF3 palmitoylation	Downregulates MYC and inhibits KRAS-mutant pancreatic cancer	Preclinical (in vivo)	FASN	[14]
TM-45	Selective TEAD palmitoylation inhibitor	Suppresses organoid growth in YAP-driven cancer	Preclinical (in vivo)	Substrate peptide	[242]
Abemaciclib	Promotes lysosomal degradation of palmitoylated B7-H4	Boosts antitumor immunity	Clinical (repurposed)	Substrate	[24]
Tunicamycin	Directly inhibits transfer of palmitate to protein	Indirectly affects the palmitoylation process	Preclinical (in vivo)	TEAD	[243]

^a^ Mechanistic tool refers to a research-use pharmacological tool.

AKT, AKT serine/threonine kinase; B7-H4, B7 homolog 4; FASN, fatty acid synthase; HCC, hepatocellular carcinoma; KRAS, Kirsten rat sarcoma viral oncogene homolog; MYC, MYC proto-oncogene, bHLH transcription factor; PCSK9, proprotein convertase subtilisin/kexin type 9; PORCN, porcupine *O*-acyltransferase; STING, stimulator of interferon genes; TEAD, transcriptional enhanced associate domain transcription factors; TKI, tyrosine kinase inhibitor; YAP, Yes-associated protein; YAP/TAZ-TEAD, Yes-associated protein/transcriptional coactivator with PDZ-binding motif–TEAD complex; YTHDF3, YTH *N*^6^-methyladenosine RNA binding protein F3 .

Taken together, current efforts are focused on elucidating DHHC structure–function relationships and identifying more selective inhibitors. These findings support the notion that small-molecule interventions targeting specific DHHCs or *S*-palmitoylation sites could reveal new therapeutic possibilities. These emerging strategies offer conceptual and practical advances in targeting dynamic *S*-palmitoylation for cancer therapy.

### Indirect modulation via lipid metabolism

Beyond PATs and APTs, FASN-catalyzed de novo synthesis of palmitate contributes to the intracellular palmitoyl–CoA pool, which varies across tissues and metabolic states [199]. Elevated FASN activity can increase endogenous palmitate production, which is converted to palmitoyl–CoA and subsequently fuels protein *S*-palmitoylation. In contrast, FASN inhibition has been shown to suppress the *S*-palmitoylation of proteins such as epidermal growth factor receptor (EGFR), PD-L1, and MYD88, affecting their localization, stability, and signaling function [200–202].

Exogenous lipid intake and metabolic disorders also affect *S*-palmitoylation through shifts in fatty acid availability. Obesity, diabetes, and other metabolic diseases are associated with lipid dysregulation and may contribute to oncogenesis. Cancer cells, in particular, show enhanced FASN-dependent fatty acid synthesis irrespective of extracellular lipid levels, supporting anabolic growth and therapeutic resistance [203–205]. Notably, lineage-plastic and therapy-resistant subtypes, such as lysine methyltransferase 2C (*KMT2C*)-deficient double-negative prostate cancer, display heightened reliance on FASN-mediated lipogenesis and are selectively vulnerable to FASN inhibition, illustrating how specific epigenetic contexts can impose differential dependence on lipid metabolism and, by extension, palmitoylation-related signaling [206]. As *S*-palmitoylation is widespread in cancers and closely tied to FASN activity, investigating the role of lipid metabolism in modulating this modification may yield further insights into cancer progression and treatment vulnerabilities.

### Targeting *S*-palmitoylation substrates and competitive inhibition strategies

In addition to enzyme-focused approaches, targeting specific *S*-palmitoylation substrates represents a promising avenue for therapeutic intervention. This includes designing small molecules or peptide mimetics that compete for *S*-palmitoylation sites, thereby preventing substrate modification (Table 6). For instance, Sun et al. [207] developed a proprotein convertase subtilisin/kexin type 9 (PCSK9)-derived peptide that competitively blocks PCSK9 *S*-palmitoylation, suppresses AKT serine/threonine kinase (AKT) activation, and enhances the antitumor effects of sorafenib in hepatocellular carcinoma. Similarly, Zhang et al. [14] engineered a YTH *N*^6^-methyladenosine RNA binding protein F3 (YTHDF3)-derived peptide that interferes with DHHC20-mediated *S*-palmitoylation, leading to down-regulation of *MYC* and inhibition of *KRAS* proto-oncogene, guanosine triphosphatase (*KRAS*)-driven pancreatic cancer progression. Beyond canonical signaling molecules, *S*-palmitoylation also modulates membrane transporters. Epidermal growth factor has been shown to enhance the *S*-palmitoylation of the organic anion transporter organic anion transporter 3, thereby increasing its trafficking to the plasma membrane and transport activity, indicating that *S*-palmitoylation can transduce growth factor signaling into altered drug and metabolite handling [208]. These substrate-based strategies expand the therapeutic toolkit for *S*-palmitoylation inhibition, opening up new possibilities for combination treatments and personalized cancer therapies.

## Conclusions and Prospects

This review primarily focuses on the pivotal role and specific mechanisms of *S*-palmitoylation as a molecular switch in mediating the transformation of cancer cells into CSCs and the transition from the normal tissue microenvironment to the TME. Unlike previously published reviews, we primarily emphasize the dynamic reversibility of *S*-palmitoylation. This reversibility provides a plausible mechanistic basis for the increased stemness, therapy resistance, immune evasion, and recurrence exhibited by tumors under conventional treatment. Undoubtedly, the reversibility of *S*-palmitoylation plays a crucial role in tumorigenesis and within the TME. While current research has largely focused on *S*-palmitoylation states mediated by palmitoyl transferases, this paper emphasizes that depalmitoylation states mediated by depalmitoylases deserve equal attention, despite the limited literature available. We propose that the imbalance of *S*-palmitoylation is the key factor in tumors developing drug resistance, metastasis, and immune evasion within the stemness or microenvironment.

*S*-palmitoylation, as a key reversible lipid modification, despite remarkable progress, still faces notable challenges in the field. Advances in mass-spectrometry-based proteomics, bioinformatics, and chemical biology, such as acyl–biotin exchange, metabolic labeling with palmitic acid analogues, and click chemistry, have expanded our capacity to detect and trace *S*-palmitoylation events in situ. Moreover, the development of *S*-palmitoylation state-specific antibodies and fluorescent probes presents new avenues for the functional dissection of this modification under both physiological and pathological conditions. However, pharmacological manipulation of *S*-palmitoylation remains in its infancy. Most DHHC inhibitors used in preclinical studies, such as 2-BP, are broad spectrum and cytotoxic. The lack of high-resolution structures for most DHHC enzymes further constrains rational drug design. Although recent findings, such as the identification of Food and Drug Administration (FDA)-approved drugs with off-target effects on DHHCs, have opened the door to drug repurposing, highly selective and low-toxicity inhibitors are still urgently needed. Alternatively, substrate-specific approaches, such as competitive peptides derived from palmitoylated motifs, have demonstrated efficacy in blocking pathogenic palmitoylation events in cancer models, offering a promising direction for precision intervention.

In conclusion, the dynamic regulation of *S*-palmitoylation represents a fertile landscape for both mechanistic exploration and therapeutic innovation. Future studies integrating structural biology, systems immunology, and medicinal chemistry are expected to unravel novel targets and strategies, including the development of selective, druggable DHHC/depalmitoylase inhibitors, the construction of context-dependent palmitoylation network maps in defined TME states, and the exploration of *S*-acylated proteins and pathway signatures as biomarkers for patient stratification and therapeutic response. Together, these efforts may ultimately lead to breakthroughs in cancer treatment through modulation of this fundamental lipid modification.

## Ethical Approval

Not applicable.

## Data Availability

Not applicable.

## References

[1] Ramazi S, Zahiri J. Posttranslational modifications in proteins: Resources, tools and prediction methods. Database. 2021;2021: Article baab012. 33826699 10.1093/database/baab012 PMC8040245

[2] Deribe YL, Pawson T, Dikic I. Post-translational modifications in signal integration. Nat Struct Mol Biol. 2010;17(6):666–672. 20495563 10.1038/nsmb.1842

[3] Zhong Q, Xiao X, Qiu Y, Xu Z, Chen C, Chong B, Zhao X, Hai S, Li S, An Z, et al. Protein posttranslational modifications in health and diseases: Functions, regulatory mechanisms, and therapeutic implications. MedComm. 2023;4(3): Article e261. 37143582 10.1002/mco2.261 PMC10152985

[4] Shao Y, Hu J, Li H, Lu K. Regulation of autophagy by protein lipidation. Adv Biotechnol. 2024;2(4):33. 10.1007/s44307-024-00040-w PMC11709147 39883197

[5] Xu M, Xu B. Protein lipidation in the tumor microenvironment: Enzymology, signaling pathways, and therapeutics. Mol Cancer. 2025;24(1):138. 40335986 10.1186/s12943-025-02309-7 PMC12057185

[6] Yuan Y, Li P, Li J, Zhao Q, Chang Y, He X. Protein lipidation in health and disease: Molecular basis, physiological function and pathological implication. Signal Transduct Target Ther. 2024;9(1):60. 38485938 10.1038/s41392-024-01759-7 PMC10940682

[7] Khoury GA, Baliban RC, Floudas CA. Proteome-wide post-translational modification statistics: Frequency analysis and curation of the swiss-prot database. Sci Rep. 2011;1(1):90. 22034591 10.1038/srep00090 PMC3201773

[8] Li M, Zhang L, Chen CW. Diverse roles of protein palmitoylation in cancer progression, immunity, stemness, and beyond. Cells. 2023;12(18):2209. 37759431 10.3390/cells12182209 PMC10526800

[9] Pan S, Chen R. Pathological implication of protein post-translational modifications in cancer. Mol Asp Med. 2022;86: Article 101097. 10.1016/j.mam.2022.101097 PMC9378605 35400524

[10] Wang Y, Yang W. Proteome-scale analysis of protein *S*-acylation comes of age. J Proteome Res. 2021;20(1):14–26. 33253586 10.1021/acs.jproteome.0c00409 PMC7775881

[11] Tewari R, West SJ, Shayahati B, Akimzhanov AM. Detection of protein S-acylation using acyl-resin assisted capture. J Vis Exp. 2020;(158). 10.3791/61016 PMC7446237 32338654

[12] Ko PJ, Dixon SJ. Protein palmitoylation and cancer. EMBO Rep. 2018;19(10): Article e46666. 30232163 10.15252/embr.201846666 PMC6172454

[13] Liu Z, Xiao M, Mo Y, Wang H, Han Y, Zhao X, Yang X, Liu Z, Xu B. Emerging roles of protein palmitoylation and its modifying enzymes in cancer cell signal transduction and cancer therapy. Int J Biol Sci. 2022;18(8):3447–3457. 35637973 10.7150/ijbs.72244 PMC9134921

[14] Zhang H, Sun Y, Wang Z, Huang X, Tang L, Jiang K, Jin X. ZDHHC20-mediated S-palmitoylation of YTHDF3 stabilizes MYC mRNA to promote pancreatic cancer progression. Nat Commun. 2024;15(1):4642. 38821916 10.1038/s41467-024-49105-3 PMC11143236

[15] Yao H, Lan J, Li C, Shi H, Brosseau JP, Wang H, Lu H, Fang C, Zhang Y, Liang L, et al. Inhibiting PD-L1 palmitoylation enhances T-cell immune responses against tumours. Nat Biomed Eng. 2019;3(4):306–317. 30952982 10.1038/s41551-019-0375-6

[16] Jiang Y, Xu Y, Zhu C, Xu G, Xu L, Rao Z, Zhou L, Jiang P, Malik S, Fang J, et al. STAT3 palmitoylation initiates a positive feedback loop that promotes the malignancy of hepatocellular carcinoma cells in mice. Sci Signal. 2023;16(814): Article eadd2282. 38051779 10.1126/scisignal.add2282 PMC10907978

[17] Fan Y, Gao Y, Nie L, Hou T, Dan W, Wang Z, Liu T, Wei Y, Wang Y, Liu B, et al. Targeting LYPLAL1-mediated cGAS depalmitoylation enhances the response to anti-tumor immunotherapy. Mol Cell. 2023;83(19):3520–3532.e7. 37802025 10.1016/j.molcel.2023.09.007

[18] Ocasio CA, Baggelaar MP, Sipthorp J, Losada de la Lastra A, Tavares M, Volaric J, Soudy C, Storck EM, Houghton JW, Palma-Duran SA, et al. A palmitoyl transferase chemical-genetic system to map ZDHHC-specific *S*-acylation. Nat Biotechnol. 2024;42(10):1548–1558. 38191663 10.1038/s41587-023-02030-0 PMC11471619

[19] Fukata Y, Fukata M. Protein palmitoylation in neuronal development and synaptic plasticity. Nat Rev Neurosci. 2010;11(3):161–175. 20168314 10.1038/nrn2788

[20] Dekker FJ, Rocks O, Vartak N, Menninger S, Hedberg C, Balamurugan R, Wetzel S, Renner S, Gerauer M, Scholermann B, et al. Small-molecule inhibition of APT1 affects Ras localization and signaling. Nat Chem Biol. 2010;6(6):449–456. 20418879 10.1038/nchembio.362

[21] Remsberg JR, Suciu RM, Zambetti NA, Hanigan TW, Firestone AJ, Inguva A, Long A, Ngo N, Lum KM, Henry CL, et al. ABHD17 regulation of plasma membrane palmitoylation and N-Ras-dependent cancer growth. Nat Chem Biol. 2021;17(8):856–864. 33927411 10.1038/s41589-021-00785-8 PMC8900659

[22] Blanc M, David FPA, van der Goot FG. SwissPalm 2: Protein *S*-palmitoylation database. Methods Mol Biol. 2019;2009:203–214. 31152406 10.1007/978-1-4939-9532-5_16

[23] Tate JG, Bamford S, Jubb HC, Sondka Z, Beare DM, Bindal N, Boutselakis H, Cole CG, Creatore C, Dawson E, et al. COSMIC: The catalogue of somatic mutations in cancer. Nucleic Acids Res. 2019;47(D1):D941–D947. 30371878 10.1093/nar/gky1015 PMC6323903

[24] Yan Y, Yu J, Wang W, Xu Y, Tison K, Xiao R, Grove S, Wei S, Vatan L, Wicha M, et al. Palmitoylation prevents B7-H4 lysosomal degradation sustaining tumor immune evasion. Nat Commun. 2025;16(1):4254. 40341398 10.1038/s41467-025-58552-5 PMC12062253

[25] Stix R, Lee CJ, Faraldo-Gomez JD, Banerjee A. Structure and mechanism of DHHC protein acyltransferases. J Mol Biol. 2020;432(18):4983–4998. 32522557 10.1016/j.jmb.2020.05.023 PMC7483407

[26] Roth AF, Feng Y, Chen L, Davis NG. The yeast DHHC cysteine-rich domain protein Akr1p is a palmitoyl transferase. J Cell Biol. 2002;159(1):23–28. 12370247 10.1083/jcb.200206120 PMC2173492

[27] Ladygina N, Martin BR, Altman A. Dynamic palmitoylation and the role of DHHC proteins in T cell activation and anergy. Adv Immunol. 2011;109:1–44. 21569911 10.1016/B978-0-12-387664-5.00001-7 PMC5464999

[28] Jennings BC, Linder ME. DHHC protein *S*-acyltransferases use similar ping-pong kinetic mechanisms but display different acyl-CoA specificities. J Biol Chem. 2012;287(10):7236–7245. 22247542 10.1074/jbc.M111.337246 PMC3293542

[29] Lan T, Delalande C, Dickinson BC. Inhibitors of DHHC family proteins. Curr Opin Chem Biol. 2021;65:118–125. 34467875 10.1016/j.cbpa.2021.07.002 PMC8671176

[30] Linder ME, Deschenes RJ. Palmitoylation: Policing protein stability and traffic. Nat Rev Mol Cell Biol. 2007;8(1):74–84. 17183362 10.1038/nrm2084

[31] Greaves J, Chamberlain LH. DHHC palmitoyl transferases: Substrate interactions and (patho)physiology. Trends Biochem Sci. 2011;36(5):245–253. 21388813 10.1016/j.tibs.2011.01.003

[32] Mitchell DA, Vasudevan A, Linder ME, Deschenes RJ. Protein palmitoylation by a family of DHHC protein *S*-acyltransferases. J Lipid Res. 2006;47(6):1118–1127. 16582420 10.1194/jlr.R600007-JLR200

[33] Rana MS, Kumar P, Lee CJ, Verardi R, Rajashankar KR, Banerjee A. Fatty acyl recognition and transfer by an integral membrane S-acyltransferase. Science. 2018;359(6372): Article eaao6326. 29326245 10.1126/science.aao6326 PMC6317078

[34] Ernst AM, Syed SA, Zaki O, Bottanelli F, Zheng H, Hacke M, Xi Z, Rivera-Molina F, Graham M, Rebane AA, et al. S-palmitoylation sorts membrane cargo for anterograde transport in the Golgi. Dev Cell. 2018;47(4):479–493.e7. 30458139 10.1016/j.devcel.2018.10.024 PMC6251505

[35] Yang Y, Hsu JM, Sun L, Chan LC, Li CW, Hsu JL, Wei Y, Xia W, Hou J, Qiu Y, et al. Palmitoylation stabilizes PD-L1 to promote breast tumor growth. Cell Res. 2019;29(1):83–86. 30514902 10.1038/s41422-018-0124-5 PMC6318320

[36] Ren JG, Xing B, Lv K, O’Keefe RA, Wu M, Wang R, Bauer KM, Ghazaryan A, Burslem GM, Zhang J, et al. RAB27B controls palmitoylation-dependent NRAS trafficking and signaling in myeloid leukemia. J Clin Invest. 2023;133(12): Article e165510. 37317963 10.1172/JCI165510 PMC10266782

[37] Tang J, Peng W, Feng Y, Le X, Wang K, Xiang Q, Li L, Wang Y, Xu C, Mu J, et al. Cancer cells escape p53’s tumor suppression through ablation of ZDHHC1-mediated p53 palmitoylation. Oncogene. 2021;40(35):5416–5426. 34282274 10.1038/s41388-021-01949-5 PMC8413129

[38] Le X, Mu J, Peng W, Tang J, Xiang Q, Tian S, Feng Y, He S, Qiu Z, Ren G, et al. DNA methylation downregulated ZDHHC1 suppresses tumor growth by altering cellular metabolism and inducing oxidative/ER stress-mediated apoptosis and pyroptosis. Theranostics. 2020;10(21):9495–9511. 32863941 10.7150/thno.45631 PMC7449911

[39] Luo CG, Gui CP, Huang GW, Chen JL, Li JY, Li PJ, Xu QH, Wang YH, Zhu JQ, Liang H, et al. Identification of ZDHHC1 as a pyroptosis inducer and potential target in the establishment of pyroptosis-related signature in localized prostate cancer. Oxidative Med Cell Longev. 2022;2022:5925817. 10.1155/2022/5925817 PMC9800907 36589680

[40] Zhang Q, Du Z, Zhou W, Li W, Yang Q, Yu H, Liu T. ZDHHC1 downregulates LIPG and inhibits colorectal cancer growth via IGF2BP1 palmitoylation. Cancer Gene Ther. 2024;31(9):1427–1437. 39069526 10.1038/s41417-024-00808-1 PMC11405259

[41] Aramsangtienchai P, Spiegelman NA, Cao J, Lin H. *S*-palmitoylation of junctional adhesion molecule C regulates its tight junction localization and cell migration. J Biol Chem. 2017;292(13):5325–5334. 28196865 10.1074/jbc.M116.730523 PMC5392678

[42] Chen B, Zheng B, DeRan M, Jarugumilli GK, Fu J, Brooks YS, Wu X. ZDHHC7-mediated S-palmitoylation of Scribble regulates cell polarity. Nat Chem Biol. 2016;12(9):686–693. 27380321 10.1038/nchembio.2119 PMC4990496

[43] Sun Y, Li X, Yin C, Zhang J, Liang E, Wu X, Ni Y, Arbesman J, Goding CR, Chen S. AMPK phosphorylates ZDHHC13 to increase MC1R activity and suppress melanomagenesis. Cancer Res. 2023;83(7):1062–1073. 36701140 10.1158/0008-5472.CAN-22-2595 PMC10073341

[44] Chen LY, Lin KR, Chen YJ, Chiang YJ, Ho KC, Shen LF, Song IW, Liu KM, Yang-Yen HF, Chen YJ, et al. Palmitoyl acyltransferase activity of ZDHHC13 regulates skin barrier development partly by controlling PADi3 and TGM1 protein stability. J Invest Dermatol. 2020;140(5):959–970.e3. 31669413 10.1016/j.jid.2019.09.017

[45] Huang J, Li J, Tang J, Wu Y, Dai F, Yi Z, Wang Y, Li Y, Wu Y, Ren G, et al. ZDHHC22-mediated mTOR palmitoylation restrains breast cancer growth and endocrine therapy resistance. Int J Biol Sci. 2022;18(7):2833–2850. 35541896 10.7150/ijbs.70544 PMC9066102

[46] Sun Y, Zhu L, Liu P, Zhang H, Guo F, Jin X. ZDHHC2-mediated AGK palmitoylation activates AKT-mTOR signaling to reduce sunitinib sensitivity in renal cell carcinoma. Cancer Res. 2023;83(12):2034–2051. 37078777 10.1158/0008-5472.CAN-22-3105 PMC10267682

[47] Wang J, Hao JW, Wang X, Guo H, Sun HH, Lai XY, Liu LY, Zhu M, Wang HY, Li YF, et al. DHHC4 and DHHC5 facilitate fatty acid uptake by palmitoylating and targeting CD36 to the plasma membrane. Cell Rep. 2019;26(1):209–221.e5. 30605677 10.1016/j.celrep.2018.12.022

[48] Sharma C, Yang W, Steen H, Freeman MR, Hemler ME. Antioxidant functions of DHHC3 suppress anti-cancer drug activities. Cell Mol Life Sci. 2021;78(5):2341–2353. 32986127 10.1007/s00018-020-03635-3 PMC8751980

[49] Sharma C, Wang HX, Li Q, Knoblich K, Reisenbichler ES, Richardson AL, Hemler ME. Protein acyltransferase DHHC3 regulates breast tumor growth, oxidative stress, and senescence. Cancer Res. 2017;77(24):6880–6890. 29055014 10.1158/0008-5472.CAN-17-1536 PMC5819883

[50] Coleman DT, Soung YH, Surh YJ, Cardelli JA, Chung J. Curcumin prevents palmitoylation of integrin beta4 in breast cancer cells. PLOS ONE. 2015;10(5): Article e0125399. 25938910 10.1371/journal.pone.0125399 PMC4418632

[51] Zhao C, Yu H, Fan X, Niu W, Fan J, Sun S, Gong M, Zhao B, Fang Z, Chen X. GSK3β palmitoylation mediated by ZDHHC4 promotes tumorigenicity of glioblastoma stem cells in temozolomide-resistant glioblastoma through the EZH2-STAT3 axis. Oncogene. 2022;11(1):28. 10.1038/s41389-022-00402-w PMC9126914 35606353

[52] Chen X, Ma H, Wang Z, Zhang S, Yang H, Fang Z. EZH2 palmitoylation mediated by ZDHHC5 in p53-mutant glioma drives malignant development and progression. Cancer Res. 2017;77(18):4998–5010. 28775165 10.1158/0008-5472.CAN-17-1139

[53] Shan J, Li X, Sun R, Yao Y, Sun Y, Kuang Q, Dai X, Sun Y. Palmitoyltransferase ZDHHC6 promotes colon tumorigenesis by targeting PPARγ-driven lipid biosynthesis via regulating lipidome metabolic reprogramming. J Exp Clin Cancer Res. 2024;43(1):227. 39148124 10.1186/s13046-024-03154-0 PMC11328492

[54] Zhou L, Lian G, Zhou T, Cai Z, Yang S, Li W, Cheng L, Ye Y, He M, Lu J, et al. Palmitoylation of GPX4 via the targetable ZDHHC8 determines ferroptosis sensitivity and antitumor immunity. Nat Cancer. 2025;6(5):768–785. 40108413 10.1038/s43018-025-00937-y

[55] Butler L, Locatelli C, Allagioti D, Lousa I, Lemonidis K, Tomkinson NCO, Salaun C, Chamberlain LH. S-acylation of Sprouty and SPRED proteins by the S-acyltransferase zDHHC17 involves a novel mode of enzyme-substrate interaction. J Biol Chem. 2023;299(1): Article 102754. 36442513 10.1016/j.jbc.2022.102754 PMC9800311

[56] Cui H, Cai X, Qian Q, Fan S, Li T, Wang T, Dai H, Song Y, Sun X, Cao P. ZDHHC11-mediated AXL palmitoylation promotes osimertinib resistance in non-small-cell lung cancer. Proc Natl Acad Sci USA. 2025;122(44): Article e2502778122. 41150710 10.1073/pnas.2502778122 PMC12595455

[57] Chen X, Hao A, Li X, Ye K, Zhao C, Yang H, Ma H, Hu L, Zhao Z, Hu L, et al. Activation of JNK and p38 MAPK mediated by ZDHHC17 drives glioblastoma m1ultiforme development and malignant progression. Theranostics. 2020;10(3):998–1015. 31938047 10.7150/thno.40076 PMC6956818

[58] Bu L, Zhang Z, Chen J, Fan Y, Guo J, Su Y, Wang H, Zhang X, Wu X, Jiang Q, et al. High-fat diet promotes liver tumorigenesis via palmitoylation and activation of AKT. Gut. 2024;73(7):1156–1168. 38191266 10.1136/gutjnl-2023-330826

[59] Chen X, Hu L, Yang H, Ma H, Ye K, Zhao C, Zhao Z, Dai H, Wang H, Fang Z. DHHC protein family targets different subsets of glioma stem cells in specific niches. J Exp Clin Cancer Res. 2019;38(1):25. 30658672 10.1186/s13046-019-1033-2 PMC6339410

[60] Du W, Zhang J, Wang Y, Li M, Cao J, Yang B, He Q, Shao X, Ying M. Palmitic acid activates c-Myc via dual palmitoylation-dependent pathways to promote colon cancer. Cell Discov. 2026;12(1):12. 41698889 10.1038/s41421-026-00869-6 PMC12909841

[61] Zhang X, Liu Y, Liao X, Wang M, Liu J, Wen Y, Liu R, Sang X, Li G, Liu P, et al. Targeting ZDHHC12-mediated PARP1 palmitoylation potentiates PARP inhibitor cytotoxicity. Cell Rep. 2026;45(2): Article 116910. 41581148 10.1016/j.celrep.2025.116910

[62] Wang Y, Chen Z, Li R, Wei D, Wang S, Luo H, Tu Y, Liu C, Xu H, Xu J, et al. S-palmitoylation of c-MET by CK2α-mediated zDHHC15 phosphorylation drives glioblastoma stem cell tumorigenicity. Neuro-Oncology. 2025;27(8):1972–1986. 40207776 10.1093/neuonc/noaf098 PMC12448808

[63] Kwon H, Choi M, Ahn Y, Jang D, Pak Y. Flotillin-1 palmitoylation turnover by APT-1 and ZDHHC-19 promotes cervical cancer progression by suppressing IGF-1 receptor desensitization and proteostasis. Cancer Gene Ther. 2023;30(2):302–312. 36257975 10.1038/s41417-022-00546-2

[64] Won SJ, Davda D, Labby KJ, Hwang SY, Pricer R, Majmudar JD, Armacost KA, Rodriguez LA, Rodriguez CL, Chong FS, et al. Molecular mechanism for isoform-selective inhibition of acyl protein thioesterases 1 and 2 (APT1 and APT2). ACS Chem Biol. 2016;11(12):3374–3382. 27748579 10.1021/acschembio.6b00720 PMC5359770

[65] Cao Y, Qiu T, Kathayat RS, Azizi SA, Thorne AK, Ahn D, Fukata Y, Fukata M, Rice PA, Dickinson BC. ABHD10 is an S-depalmitoylase affecting redox homeostasis through peroxiredoxin-5. Nat Chem Biol. 2019;15(12):1232–1240. 31740833 10.1038/s41589-019-0399-y PMC6871660

[66] Ni H, Wang Y, Yao K, Wang L, Huang J, Xiao Y, Chen H, Liu B, Yang CY, Zhao J. Cyclical palmitoylation regulates TLR9 signalling and systemic autoimmunity in mice. Nat Commun. 2024;15(1):1. 38169466 10.1038/s41467-023-43650-z PMC10762000

[67] Won SJ, Cheung See Kit M, Martin BR. Protein depalmitoylases. Crit Rev Biochem Mol Biol. 2018;53(1):83–98. 29239216 10.1080/10409238.2017.1409191 PMC6009847

[68] Azizi SA, Kathayat RS, Dickinson BC. Activity-based sensing of *S*-depalmitoylases: Chemical technologies and biological discovery. Acc Chem Res. 2019;52(11):3029–3038. 31577124 10.1021/acs.accounts.9b00354 PMC7201403

[69] Lin DT, Conibear E. ABHD17 proteins are novel protein depalmitoylases that regulate N-Ras palmitate turnover and subcellular localization. eLife. 2015;4: Article e11306. 26701913 10.7554/eLife.11306 PMC4755737

[70] Rauwerdink A, Kazlauskas RJ. How the same core catalytic machinery catalyzes 17 different reactions: The serine-histidine-aspartate catalytic triad of α/β-hydrolase fold enzymes. ACS Catal. 2015;5(10):6153–6176. 28580193 10.1021/acscatal.5b01539 PMC5455348

[71] Bellizzi JJ 3rd, Widom J, Kemp C, Lu JY, Das AK, Hofmann SL, Clardy J. The crystal structure of palmitoyl protein thioesterase 1 and the molecular basis of infantile neuronal ceroid lipofuscinosis. Proc Natl Acad Sci USA. 2000;97(9):4573–4578. 10781062 10.1073/pnas.080508097 PMC18274

[72] Rebecca VW, Nicastri MC, Fennelly C, Chude CI, Barber-Rotenberg JS, Ronghe A, McAfee Q, McLaughlin NP, Zhang G, Goldman AR, et al. PPT1 promotes tumor growth and is the molecular target of chloroquine derivatives in cancer. Cancer Discov. 2019;9(2):220–229. 30442709 10.1158/2159-8290.CD-18-0706 PMC6368875

[73] Luo Q, Hu S, Tang Y, Yang D, Chen Q. PPT1 promotes growth and inhibits ferroptosis of oral squamous cell carcinoma cells. Curr Cancer Drug Targets. 2024;24(10):1047–1060. 38299399 10.2174/0115680096294098240123104657

[74] Zhu Z, Feng S, Zeng A, Song L. Advances in palmitoylation: A key regulator of liver cancer development and therapeutic targets. Biochem Pharmacol. 2025;234: Article 116810. 39978688 10.1016/j.bcp.2025.116810

[75] Vesa J, Hellsten E, Verkruyse LA, Camp LA, Rapola J, Santavuori P, Hofmann SL, Peltonen L. Mutations in the palmitoyl protein thioesterase gene causing infantile neuronal ceroid lipofuscinosis. Nature. 1995;376(6541):584–587. 7637805 10.1038/376584a0

[76] Gorenberg EL, Massaro Tieze S, Yucel B, Zhao HR, Chou V, Wirak GS, Tomita S, Lam TT, Chandra SS. Identification of substrates of palmitoyl protein thioesterase 1 highlights roles of depalmitoylation in disulfide bond formation and synaptic function. PLOS Biol. 2022;20(3): Article e3001590. 35358180 10.1371/journal.pbio.3001590 PMC9004782

[77] Bekker-Jensen DB, Kelstrup CD, Batth TS, Larsen SC, Haldrup C, Bramsen JB, Sorensen KD, Hoyer S, Orntoft TF, Andersen CL, et al. An optimized shotgun strategy for the rapid generation of comprehensive human proteomes. Cell Syst. 2017;4(6):587–599.e4. 28601559 10.1016/j.cels.2017.05.009 PMC5493283

[78] Hein MY, Hubner NC, Poser I, Cox J, Nagaraj N, Toyoda Y, Gak IA, Weisswange I, Mansfeld J, Buchholz F, et al. A human interactome in three quantitative dimensions organized by stoichiometries and abundances. Cell. 2015;163(3):712–723. 26496610 10.1016/j.cell.2015.09.053

[79] Loh JJ, Ma S. Hallmarks of cancer stemness. Cell Stem Cell. 2024;31(5):617–639. 38701757 10.1016/j.stem.2024.04.004

[80] Tabata K, Imai K, Fukuda K, Yamamoto K, Kunugi H, Fujita T, Kaminishi T, Tischer C, Neumann B, Reither S, et al. Palmitoylation of ULK1 by ZDHHC13 plays a crucial role in autophagy. Nat Commun. 2024;15(1):7194. 39169022 10.1038/s41467-024-51402-w PMC11339336

[81] Yu F, Qian Z. Mechanisms for regulation of RAS palmitoylation and plasma membrane trafficking in hematopoietic malignancies. J Clin Invest. 2023;133(12): Article e171104. 37317974 10.1172/JCI171104 PMC10266771

[82] Li W, Liu J, Yu T, Lu F, Miao Q, Meng X, Xiao W, Yang H, Zhang X. ZDHHC9-mediated Bip/GRP78 S-palmitoylation inhibits unfolded protein response and promotes bladder cancer progression. Cancer Lett. 2024;598: Article 217118. 39002690 10.1016/j.canlet.2024.217118

[83] Lin Z, Agarwal S, Tan S, Shi H, Lu X, Tao Z, Dong X, Wu X, Zhao JC, Yu J. Palmitoyl acyltransferase ZDHHC7 inhibits androgen receptor and suppresses prostate cancer. Oncogene. 2023;42(26):2126–2138. 37198397 10.1038/s41388-023-02718-2

[84] Zhang Z, Li X, Yang F, Chen C, Liu P, Ren Y, Sun P, Wang Z, You Y, Zeng YX, et al. DHHC9-mediated GLUT1 S-palmitoylation promotes glioblastoma glycolysis and tumorigenesis. Nat Commun. 2021;12(1):5872. 34620861 10.1038/s41467-021-26180-4 PMC8497546

[85] Pei X, Li KY, Shen Y, Li JT, Lei MZ, Fang CY, Lu HJ, Yang HJ, Wen W, Yin M, et al. Palmitoylation of MDH2 by ZDHHC18 activates mitochondrial respiration and accelerates ovarian cancer growth. Sci China Life Sci. 2022;65(10):2017–2030. 35366151 10.1007/s11427-021-2048-2

[86] Zhou B, Wang Y, Zhang L, Shi X, Kong H, Zhang M, Liu Y, Shao X, Liu Z, Song H, et al. The palmitoylation of AEG-1 dynamically modulates the progression of hepatocellular carcinoma. Theranostics. 2022;12(16):6898–6914. 36276642 10.7150/thno.78377 PMC9576614

[87] Liu B, Zhao X, Zhang S, Li Q, Li X, Huang D, Xia J, Ma N, Duan Y, Zhang X, et al. Targeting ZDHHC21/FASN axis for the treatment of diffuse large B-cell lymphoma. Leukemia. 2024;38(2):351–364. 38195819 10.1038/s41375-023-02130-5 PMC10844076

[88] Najafi M, Farhood B, Mortezaee K. Cancer stem cells (CSCs) in cancer progression and therapy. J Cell Physiol. 2019;234(6):8381–8395. 30417375 10.1002/jcp.27740

[89] Clevers H, Nusse R. Wnt/β-catenin signaling and disease. Cell. 2012;149(6):1192–1205. 22682243 10.1016/j.cell.2012.05.012

[90] Reya T, Clevers H. Wnt signalling in stem cells and cancer. Nature. 2005;434(7035):843–850. 15829953 10.1038/nature03319

[91] Takada R, Satomi Y, Kurata T, Ueno N, Norioka S, Kondoh H, Takao T, Takada S. Monounsaturated fatty acid modification of Wnt protein: Its role in Wnt secretion. Dev Cell. 2006;11(6):791–801. 17141155 10.1016/j.devcel.2006.10.003

[92] Liu Y, Qi X, Donnelly L, Elghobashi-Meinhardt N, Long T, Zhou RW, Sun Y, Wang B, Li X. Mechanisms and inhibition of porcupine-mediated Wnt acylation. Nature. 2022;607(7920):816–822. 35831507 10.1038/s41586-022-04952-2 PMC9404457

[93] Zheng S, Lin J, Pang Z, Zhang H, Wang Y, Ma L, Zhang H, Zhang X, Chen M, Zhang X, et al. Aberrant cholesterol metabolism and Wnt/β-catenin signaling coalesce via Frizzled5 in supporting cancer growth. Adv Sci. 2022;9(28): Article e2200750. 10.1002/advs.202200750 PMC9534957 35975457

[94] Teo S, Bossio A, Stamatakou E, Pascual-Vargas P, Jones ME, Schuhmacher LN, Salinas PC. S-acylation of the Wnt receptor Frizzled-5 by zDHHC5 controls its cellular localization and synaptogenic activity in the rodent hippocampus. Dev Cell. 2023;58(20):2063–2079.e9. 37557176 10.1016/j.devcel.2023.07.012

[95] Tang Q, Chen J, Di Z, Yuan W, Zhou Z, Liu Z, Han S, Liu Y, Ying G, Shu X, et al. TM4SF1 promotes EMT and cancer stemness via the Wnt/β-catenin/SOX2 pathway in colorectal cancer. J Exp Clin Cancer Res. 2020;39(1):232. 33153498 10.1186/s13046-020-01690-z PMC7643364

[96] Lin X, Wang F, Chen J, Liu J, Lin YB, Li L, Chen CB, Xu Q. *N^6^*-methyladenosine modification of CENPK mRNA by ZC3H13 promotes cervical cancer stemness and chemoresistance. Mil Med Res. 2022;9(1):19. 35418160 10.1186/s40779-022-00378-z PMC9008995

[97] Wang Q, Liang N, Yang T, Li Y, Li J, Huang Q, Wu C, Sun L, Zhou X, Cheng X, et al. DNMT1-mediated methylation of BEX1 regulates stemness and tumorigenicity in liver cancer. J Hepatol. 2021;75(5):1142–1153. 34217777 10.1016/j.jhep.2021.06.025

[98] Zhang W, Ruan X, Li Y, Zhi J, Hu L, Hou X, Shi X, Wang X, Wang J, Ma W, et al. KDM1A promotes thyroid cancer progression and maintains stemness through the Wnt/β-catenin signaling pathway. Theranostics. 2022;12(4):1500–1517. 35198054 10.7150/thno.66142 PMC8825597

[99] Zhu GQ, Wang Y, Wang B, Liu WR, Dong SS, Chen EB, Cai JL, Wan JL, Du JX, Song LN, et al. Targeting HNRNPM inhibits cancer stemness and enhances antitumor immunity in Wnt-activated hepatocellular carcinoma. Cell Mol Gastroenterol Hepatol. 2022;13(5):1413–1447. 35158098 10.1016/j.jcmgh.2022.02.006 PMC8938476

[100] Harvey K, Tapon N. The Salvador-Warts-Hippo pathway – An emerging tumour-suppressor network. Nat Rev Cancer. 2007;7(3):182–191. 17318211 10.1038/nrc2070

[101] Zhao B, Wei X, Li W, Udan RS, Yang Q, Kim J, Xie J, Ikenoue T, Yu J, Li L, et al. Inactivation of YAP oncoprotein by the Hippo pathway is involved in cell contact inhibition and tissue growth control. Genes Dev. 2007;21(21):2747–2761. 17974916 10.1101/gad.1602907 PMC2045129

[102] Zhao B, Li L, Lei Q, Guan KL. The Hippo-YAP pathway in organ size control and tumorigenesis: An updated version. Genes Dev. 2010;24(9):862–874. 20439427 10.1101/gad.1909210 PMC2861185

[103] Hansen CG, Moroishi T, Guan KL. YAP and TAZ: A nexus for Hippo signaling and beyond. Trends Cell Biol. 2015;25(9):499–513. 26045258 10.1016/j.tcb.2015.05.002 PMC4554827

[104] Davis JR, Tapon N. Hippo signalling during development. Development. 2019;146(18): Article dev167106. 31527062 10.1242/dev.167106 PMC7100553

[105] Franklin JM, Wu Z, Guan KL. Insights into recent findings and clinical application of YAP and TAZ in cancer. Nat Rev Cancer. 2023;23(8):512–525. 37308716 10.1038/s41568-023-00579-1

[106] Vici P, Ercolani C, Di Benedetto A, Pizzuti L, Di Lauro L, Sperati F, Terrenato I, Gamucci T, Natoli C, Di Filippo F, et al. Topographic expression of the Hippo transducers TAZ and YAP in triple-negative breast cancer treated with neoadjuvant chemotherapy. J Exp Clin Cancer Res. 2016;35(1):62. 27039292 10.1186/s13046-016-0338-7 PMC4818869

[107] Tang TT, Konradi AW, Feng Y, Peng X, Ma M, Li J, Yu FX, Guan KL, Post L. Small molecule inhibitors of TEAD auto-palmitoylation selectively inhibit proliferation and tumor growth of NF2-deficient mesothelioma. Mol Cancer Ther. 2021;20(6):986–998. 33850002 10.1158/1535-7163.MCT-20-0717

[108] Sun Y, Hu L, Tao Z, Jarugumilli GK, Erb H, Singh A, Li Q, Cotton JL, Greninger P, Egan RK, et al. Pharmacological blockade of TEAD-YAP reveals its therapeutic limitation in cancer cells. Nat Commun. 2022;13(1):6744. 36347861 10.1038/s41467-022-34559-0 PMC9643419

[109] Zhou Y, Zhang C, Xia J, Shang S, Liu Q, Guo X, Zhang J, Cui S, Wang X, Liu R, et al. ZDHHC14-mediated TEAD4 palmitoylation drives Th17 cell recruitment in renal immunopathology. Research. 2025;8:0954. 41112095 10.34133/research.0954 PMC12529098

[110] Frum T, Watts JL, Ralston A. YAP1 and WWTR1 prevent the premature onset of pluripotency prior to the 16-cell stage. Development. 2019;146(17): Article dev179861. 31444221 10.1242/dev.179861 PMC6765126

[111] Zou H, Luo J, Guo Y, Deng L, Zeng L, Pan Y, Li P. Tyrosine phosphorylation-mediated YAP1-TFAP2A interactions coordinate transcription and trastuzumab resistance in HER2+ breast cancer. Drug Resist Updat. 2024;73: Article 101051. 38219531 10.1016/j.drup.2024.101051

[112] Voytyuk O, Ohata Y, Moustakas A, Ten Dijke P, Heldin CH. Smad7 palmitoylation by the S-acyltransferase zDHHC17 enhances its inhibitory effect on TGF-β/Smad signaling. J Biol Chem. 2024;300(7): Article 107462. 38876303 10.1016/j.jbc.2024.107462 PMC11277750

[113] Peng D, Fu M, Wang M, Wei Y, Wei X. Targeting TGF-β signal transduction for fibrosis and cancer therapy. Mol Cancer. 2022;21(1):104. 35461253 10.1186/s12943-022-01569-x PMC9033932

[114] Wu M, Wu S, Chen W, Li YP. The roles and regulatory mechanisms of TGF-β and BMP signaling in bone and cartilage development, homeostasis and disease. Cell Res. 2024;34(2):101–123. 38267638 10.1038/s41422-023-00918-9 PMC10837209

[115] Liang S, Zheng R, Zuo B, Li J, Wang Y, Han Y, Dong H, Zhao X, Zhang Y, Wang P, et al. SMAD7 expression in CAR-T cells improves persistence and safety for solid tumors. Cell Mol Immunol. 2024;21(3):213–226. 38177245 10.1038/s41423-023-01120-y PMC10901810

[116] Fan X, Fan J, Yang H, Zhao C, Niu W, Fang Z, Chen X. Heterogeneity of subsets in glioblastoma mediated by Smad3 palmitoylation. Oncogene. 2021;10(10):72. 10.1038/s41389-021-00361-8 PMC8551152 34707087

[117] Chen QT, Zhang ZY, Huang QL, Chen HZ, Hong WB, Lin T, Zhao WX, Wang XM, Ju CY, Wu LZ, et al. HK1 from hepatic stellate cell-derived extracellular vesicles promotes progression of hepatocellular carcinoma. Nat Metab. 2022;4(10):1306–1321. 36192599 10.1038/s42255-022-00642-5 PMC9584821

[118] Li W, Li W, Zou L, Ji S, Li C, Liu K, Zhang G, Sun Q, Xiao F, Chen D. Membrane targeting of inhibitory Smads through palmitoylation controls TGF-β/BMP signaling. Proc Natl Acad Sci USA. 2017;114(50):13206–13211. 29180412 10.1073/pnas.1710540114 PMC5740658

[119] Wegleiter T, Buthey K, Gonzalez-Bohorquez D, Hruzova M, Bin Imtiaz MK, Abegg A, Mebert I, Molteni A, Kollegger D, Pelczar P, et al. Palmitoylation of BMPR1a regulates neural stem cell fate. Proc Natl Acad Sci USA. 2019;116(51):25688–25696. 31772009 10.1073/pnas.1912671116 PMC6926058

[120] Briscoe J, Therond PP. The mechanisms of Hedgehog signalling and its roles in development and disease. Nat Rev Mol Cell Biol. 2013;14(7):416–429. 23719536 10.1038/nrm3598

[121] Metcalfe C, Siebel CW. The Hedgehog hold on homeostasis. Cell Stem Cell. 2015;17(5):505–506. 26544111 10.1016/j.stem.2015.10.010

[122] Ingham PW, McMahon AP. Hedgehog signaling in animal development: Paradigms and principles. Genes Dev. 2001;15(23):3059–3087. 11731473 10.1101/gad.938601

[123] Chamoun Z, Mann RK, Nellen D, von Kessler DP, Bellotto M, Beachy PA, Basler K. Skinny hedgehog, an acyltransferase required for palmitoylation and activity of the hedgehog signal. Science. 2001;293(5537):2080–2084. 11486055 10.1126/science.1064437

[124] Pepinsky RB, Zeng C, Wen D, Rayhorn P, Baker DP, Williams KP, Bixler SA, Ambrose CM, Garber EA, Miatkowski K, et al. Identification of a palmitic acid-modified form of human Sonic hedgehog. J Biol Chem. 1998;273(22):14037–14045. 9593755 10.1074/jbc.273.22.14037

[125] Chen MH, Li YJ, Kawakami T, Xu SM, Chuang PT. Palmitoylation is required for the production of a soluble multimeric Hedgehog protein complex and long-range signaling in vertebrates. Genes Dev. 2004;18(6):641–659. 15075292 10.1101/gad.1185804 PMC387240

[126] Varjosalo M, Taipale J. Hedgehog: Functions and mechanisms. Genes Dev. 2008;22(18):2454–2472. 18794343 10.1101/gad.1693608

[127] Chen J, Zhu Y, Zhao D, Zhang L, Zhang J, Xiao Y, Wu Q, Wang Y, Zhan Q. Co-targeting FAK and Gli1 inhibits the tumor-associated macrophages-released CCL22-mediated esophageal squamous cell carcinoma malignancy. MedComm. 2023;4(6): Article e381. 37846367 10.1002/mco2.381 PMC10576977

[128] Beachy PA, Karhadkar SS, Berman DM. Tissue repair and stem cell renewal in carcinogenesis. Nature. 2004;432(7015):324–331. 15549094 10.1038/nature03100

[129] Gu Y, Wang Y, He L, Zhang J, Zhu X, Liu N, Wang J, Lu T, He L, Tian Y, et al. Circular RNA circIPO11 drives self-renewal of liver cancer initiating cells via Hedgehog signaling. Mol Cancer. 2021;20(1):132. 34649567 10.1186/s12943-021-01435-2 PMC8515748

[130] Zhang Z, Yang J, Liu R, Ma J, Wang K, Wang X, Tang N. Inhibiting HMGCR represses stemness and metastasis of hepatocellular carcinoma via Hedgehog signaling. Genes Dis. 2024;11(5): Article 101285. 39022130 10.1016/j.gendis.2024.101285 PMC11252768

[131] Ayers KL, Therond PP. Evaluating Smoothened as a G-protein-coupled receptor for Hedgehog signalling. Trends Cell Biol. 2010;20(5):287–298. 20207148 10.1016/j.tcb.2010.02.002

[132] Cheng LH, Hsu CC, Tsai HW, Liao WY, Yang PM, Liao TY, Hsieh HY, Chan TS, Tsai KK. ASPM activates Hedgehog and Wnt signaling to promote small cell lung cancer stemness and progression. Cancer Res. 2023;83(6):830–844. 36638332 10.1158/0008-5472.CAN-22-2496

[133] Degirmenci B, Valenta T, Dimitrieva S, Hausmann G, Basler K. GLI1-expressing mesenchymal cells form the essential Wnt-secreting niche for colon stem cells. Nature. 2018;558(7710):449–453. 29875413 10.1038/s41586-018-0190-3

[134] Quail DF, Joyce JA. Microenvironmental regulation of tumor progression and metastasis. Nat Med. 2013;19(11):1423–1437. 24202395 10.1038/nm.3394 PMC3954707

[135] Chen Z, Han F, Du Y, Shi H, Zhou W. Hypoxic microenvironment in cancer: Molecular mechanisms and therapeutic interventions. Signal Transduct Target Ther. 2023;8(1):70. 36797231 10.1038/s41392-023-01332-8 PMC9935926

[136] Semenza GL. Targeting HIF-1 for cancer therapy. Nat Rev Cancer. 2003;3(10):721–732. 13130303 10.1038/nrc1187

[137] Morant-Ferrando B, Jimenez-Blasco D, Alonso-Batan P, Agulla J, Lapresa R, Garcia-Rodriguez D, Yunta-Sanchez S, Lopez-Fabuel I, Fernandez E, Carmeliet P, et al. Fatty acid oxidation organizes mitochondrial supercomplexes to sustain astrocytic ROS and cognition. Nat Metab. 2023;5(8):1290–1302. 37460843 10.1038/s42255-023-00835-6 PMC10447235

[138] Liang Z, He H, Zhang B, Kai Z, Zong L. Hypoxia expedites the progression of papillary thyroid carcinoma by promoting the CPT1A-mediated fatty acid oxidative pathway. Drug Dev Res. 2024;85(2): Article e22168. 38450796 10.1002/ddr.22168

[139] Wei F, Wang Y, Yao J, Mei L, Huang X, Kong H, Chen J, Chen X, Liu L, Wang Z, et al. ZDHHC7-mediated *S*-palmitoylation of ATG16L1 facilitates LC3 lipidation and autophagosome formation. Autophagy. 2024;20(12):2719–2737. 39087410 10.1080/15548627.2024.2386915 PMC11587844

[140] Xia F, Li W, Wang W, Liu J, Li X, Cai J, Shan H, Cai Z, Cui J. S-palmitoylation coordinates the trafficking of ATG9A to mediate autophagy initiation. Autophagy. 2025;21(11):2422–2442. 40394978 10.1080/15548627.2025.2509376 PMC12674483

[141] Liu Y, Zhang H, Liu Y, Zhang S, Su P, Wang L, Li Y, Liang Y, Wang X, Zhao W, et al. Hypoxia-induced GPCPD1 depalmitoylation triggers mitophagy via regulating PRKN-mediated ubiquitination of VDAC1. Autophagy. 2023;19(9):2443–2463. 36803235 10.1080/15548627.2023.2182482 PMC10392732

[142] Boucher JM, Clark RP, Chong DC, Citrin KM, Wylie LA, Bautch VL. Dynamic alterations in decoy VEGF receptor-1 stability regulate angiogenesis. Nat Commun. 2017;8(1):15699. 28589930 10.1038/ncomms15699 PMC5467243

[143] Zhang M, Zhou L, Xu Y, Yang M, Xu Y, Komaniecki GP, Kosciuk T, Chen X, Lu X, Zou X, et al. A STAT3 palmitoylation cycle promotes T_H_17 differentiation and colitis. Nature. 2020;586(7829):434–439. 33029007 10.1038/s41586-020-2799-2 PMC7874492

[144] Chen DS, Mellman I. Elements of cancer immunity and the cancer-immune set point. Nature. 2017;541(7637):321–330. 28102259 10.1038/nature21349

[145] Binnewies M, Roberts EW, Kersten K, Chan V, Fearon DF, Merad M, Coussens LM, Gabrilovich DI, Ostrand-Rosenberg S, Hedrick CC, et al. Understanding the tumor immune microenvironment (TIME) for effective therapy. Nat Med. 2018;24(5):541–550. 29686425 10.1038/s41591-018-0014-x PMC5998822

[146] Joyce JA, Fearon DT. T cell exclusion, immune privilege, and the tumor microenvironment. Science. 2015;348(6230):74–80. 25838376 10.1126/science.aaa6204

[147] Han M, Lv Y, Chen Y, Li Z, Tian J, Zhou H, Wang Y, Su W, Zhong J. Advances in targeting protein S-palmitoylation in tumor immunity and therapy. Front Oncol. 2025;15:1547636. 40066091 10.3389/fonc.2025.1547636 PMC11891048

[148] Burr ML, Sparbier CE, Chan YC, Williamson JC, Woods K, Beavis PA, Lam EYN, Henderson MA, Bell CC, Stolzenburg S, et al. CMTM6 maintains the expression of PD-L1 and regulates anti-tumour immunity. Nature. 2017;549(7670):101–105. 28813417 10.1038/nature23643 PMC5706633

[149] Mezzadra R, Sun C, Jae LT, Gomez-Eerland R, de Vries E, Wu W, Logtenberg MEW, Slagter M, Rozeman EA, Hofland I, et al. Identification of CMTM6 and CMTM4 as PD-L1 protein regulators. Nature. 2017;549(7670):106–110. 28813410 10.1038/nature23669 PMC6333292

[150] Wang Q, Wang J, Yu D, Zhang Q, Hu H, Xu M, Zhang H, Tian S, Zheng G, Lu D, et al. Benzosceptrin C induces lysosomal degradation of PD-L1 and promotes antitumor immunity by targeting DHHC3. Cell Rep Med. 2024;5(2): Article 101357. 38237597 10.1016/j.xcrm.2023.101357 PMC10897506

[151] Wang J, Wang Y, Jiang X, Xu M, Wang M, Wang R, Zheng B, Chen M, Ke Q, Long J. Unleashing the power of immune checkpoints: Post-translational modification of novel molecules and clinical applications. Cancer Lett. 2024;588: Article 216758. 38401885 10.1016/j.canlet.2024.216758

[152] Luo X, Bao X, Weng X, Bai X, Feng Y, Huang J, Liu S, Jia H, Yu B. The protective effect of quercetin on macrophage pyroptosis via TLR2/Myd88/NF-κB and ROS/AMPK pathway. Life Sci. 2022;291: Article 120064. 34688696 10.1016/j.lfs.2021.120064

[153] Cai H, Yan L, Liu N, Xu M, Cai H. IFI16 promotes cervical cancer progression by upregulating PD-L1 in immunomicroenvironment through STING-TBK1-NF-kB pathway. Biomed Pharmacother. 2020;123: Article 109790. 31896065 10.1016/j.biopha.2019.109790

[154] Leishman S, Aljadeed NM, Qian L, Cockcroft S, Behmoaras J, Anand PK. Fatty acid synthesis promotes inflammasome activation through NLRP3 palmitoylation. Cell Rep. 2024;43(8): Article 114516. 39024103 10.1016/j.celrep.2024.114516

[155] Sun J, Wang P, Yi Z, Wu Y, Wei Y, Fang H, Song D, Chen Y, Du H, Huang J, et al. Blocking WNT7A enhances MHC-I antigen presentation and enhances the effectiveness of immune checkpoint blockade therapy. Cancer Immunol Res. 2025;13(3):400–416. 39602462 10.1158/2326-6066.CIR-24-0484 PMC11876963

[156] Dong W, He B, Cao Y, Yang R, Zhang S, Kong Y, Lu D, Zheng X, Hou Y, Zhu M, et al. Low-dose SAHA enhances CD8^+^ T cell-mediated antitumor immunity by boosting MHC I expression in non-small cell lung cancer. Cell Oncol. 2025;48(1):249–264. 10.1007/s13402-024-00989-9 PMC11850570 39283477

[157] Dong H, Wen C, He L, Zhang J, Xiang N, Liang L, Hu L, Li W, Liu J, Shi M, et al. Nilotinib boosts the efficacy of anti-PDL1 therapy in colorectal cancer by restoring the expression of MHC-I. J Transl Med. 2024;22(1):769. 39143573 10.1186/s12967-024-05572-2 PMC11325812

[158] Huang J, Tsang WY, Fang XN, Zhang Y, Luo J, Gong LQ, Zhang BF, Wong CN, Li ZH, Liu BL, et al. FASN inhibition decreases MHC-I degradation and synergizes with PD-L1 checkpoint blockade in hepatocellular carcinoma. Cancer Res. 2024;84(6):855–871. 38486485 10.1158/0008-5472.CAN-23-0966

[159] Du W, Hua F, Li X, Zhang J, Li S, Wang W, Zhou J, Wang W, Liao P, Yan Y, et al. Loss of optineurin drives cancer immune evasion via palmitoylation-dependent IFNGR1 lysosomal sorting and degradation. Cancer Discov. 2021;11(7):1826–1843. 33627378 10.1158/2159-8290.CD-20-1571 PMC8292167

[160] Ren J, Li N, Pei S, Lian Y, Li L, Peng Y, Liu Q, Guo J, Wang X, Han Y, et al. Histone methyltransferase WHSC1 loss dampens MHC-I antigen presentation pathway to impair IFN-γ-stimulated antitumor immunity. J Clin Invest. 2022;132(8): Article e153167. 35230972 10.1172/JCI153167 PMC9012282

[161] Seth RB, Sun L, Ea CK, Chen ZJ. Identification and characterization of MAVS, a mitochondrial antiviral signaling protein that activates NF-κB and IRF3. Cell. 2005;122(5):669–682. 16125763 10.1016/j.cell.2005.08.012

[162] Liu J, Zhou J, Luan Y, Li X, Meng X, Liao W, Tang J, Wang Z. cGAS-STING, inflammasomes and pyroptosis: An overview of crosstalk mechanism of activation and regulation. Cell Commun Signal. 2024;22(1):22. 38195584 10.1186/s12964-023-01466-w PMC10775518

[163] Chen Q, Sun L, Chen ZJ. Regulation and function of the cGAS-STING pathway of cytosolic DNA sensing. Nat Immunol. 2016;17(10):1142–1149. 27648547 10.1038/ni.3558

[164] Su C, Cheng T, Huang J, Zhang T, Yin H. 4-Octyl itaconate restricts STING activation by blocking its palmitoylation. Cell Rep. 2023;42(9): Article 113040. 37624697 10.1016/j.celrep.2023.113040

[165] Wang L, Li M, Lian G, Yang S, Cai J, Cai Z, Wu Y, Cui J. Palmitoylation acts as a checkpoint for MAVS aggregation to promote antiviral innate immune responses. J Clin Invest. 2024;134(23): Article e177924. 39621307 10.1172/JCI177924 PMC11601910

[166] Dai J, Su Y, Zhong S, Cong L, Liu B, Yang J, Tao Y, He Z, Chen C, Jiang Y. Exosomes: Key players in cancer and potential therapeutic strategy. Signal Transduct Target Ther. 2020;5(1):145. 32759948 10.1038/s41392-020-00261-0 PMC7406508

[167] Whiteside TL. Exosomes and tumor-mediated immune suppression. J Clin Invest. 2016;126(4):1216–1223. 26927673 10.1172/JCI81136 PMC4811135

[168] Zhang HG, Grizzle WE. Exosomes and cancer: A newly described pathway of immune suppression. Clin Cancer Res. 2011;17(5):959–964. 21224375 10.1158/1078-0432.CCR-10-1489 PMC3155407

[169] Qu M, Liu X, Wang X, Li Z, Zhou L, Li H. Palmitoylation of vacuole membrane protein 1 promotes small extracellular vesicle secretion via interaction with ALIX and influences intercellular communication. Cell Commun Signal. 2024;22(1):150. 38403678 10.1186/s12964-024-01529-6 PMC10895845

[170] Shi Y, Du L, Lv D, Li H, Shang J, Lu J, Zhou L, Bai L, Tang H. Exosomal interferon-induced transmembrane protein 2 transmitted to dendritic cells inhibits interferon alpha pathway activation and blocks anti-hepatitis B virus efficacy of exogenous interferon alpha. Hepatology. 2019;69(6):2396–2413. 30723923 10.1002/hep.30548 PMC6593428

[171] Balasubramanian A, Hsu AY, Ghimire L, Tahir M, Devant P, Fontana P, Du G, Liu X, Fabin D, Kambara H, et al. The palmitoylation of gasdermin D directs its membrane translocation and pore formation during pyroptosis. Sci Immunol. 2024;9(94): Article eadn1452. 38530158 10.1126/sciimmunol.adn1452 PMC11367861

[172] Yu T, Hou D, Zhao J, Lu X, Greentree WK, Zhao Q, Yang M, Conde DG, Linder ME, Lin H. NLRP3 Cys126 palmitoylation by ZDHHC7 promotes inflammasome activation. Cell Rep. 2024;43(4): Article 114070. 38583156 10.1016/j.celrep.2024.114070 PMC11130711

[173] Ganbold M, Owada Y, Ozawa Y, Shimamoto Y, Ferdousi F, Tominaga K, Zheng YW, Ohkohchi N, Isoda H. Isorhamnetin alleviates steatosis and fibrosis in mice with nonalcoholic steatohepatitis. Sci Rep. 2019;9(1):16210. 31700054 10.1038/s41598-019-52736-y PMC6838085

[174] Evavold CL, Ruan J, Tan Y, Xia S, Wu H, Kagan JC. The pore-forming protein gasdermin D regulates interleukin-1 secretion from living macrophages. Immunity. 2018;48(1):35–44.e6. 29195811 10.1016/j.immuni.2017.11.013 PMC5773350

[175] Toldo S, Abbate A. The role of the NLRP3 inflammasome and pyroptosis in cardiovascular diseases. Nat Rev Cardiol. 2024;21(4):219–237. 37923829 10.1038/s41569-023-00946-3 PMC11550901

[176] Vanauberg D, Schulz C, Lefebvre T. Involvement of the pro-oncogenic enzyme fatty acid synthase in the hallmarks of cancer: A promising target in anti-cancer therapies. Oncogene. 2023;12(1):16. 10.1038/s41389-023-00460-8 PMC10024702 36934087

[177] Montgomery DC, Sorum AW, Guasch L, Nicklaus MC, Meier JL. Metabolic regulation of histone acetyltransferases by endogenous acyl-CoA cofactors. Chem Biol. 2015;22(8):1030–1039. 26190825 10.1016/j.chembiol.2015.06.015 PMC4546520

[178] Liu X, He H, Qi M, Jiang Z, Lin B, Wang X, Wang D, Ma M, Jiang W, Zhou R. A small molecule directly targets NLRP3 to promote inflammasome activation and antitumor immunity. Cell Death Dis. 2025;16(1):252. 40185713 10.1038/s41419-025-07578-0 PMC11971322

[179] Shalapour S, Karin M. Immunity, inflammation, and cancer: An eternal fight between good and evil. J Clin Invest. 2015;125(9):3347–3355. 26325032 10.1172/JCI80007 PMC4588298

[180] Karki R, Kanneganti TD. Diverging inflammasome signals in tumorigenesis and potential targeting. Nat Rev Cancer. 2019;19(4):197–214. 30842595 10.1038/s41568-019-0123-y PMC6953422

[181] Gu H, Deng W, Zhang Y, Chang Y, Shelat VG, Tsuchida K, Lino-Silva LS, Wang Z. NLRP3 activation in tumor-associated macrophages enhances lung metastasis of pancreatic ductal adenocarcinoma. Transl Lung Cancer Res. 2022;11(5):858–868. 35693281 10.21037/tlcr-22-311 PMC9186165

[182] Yingze Y, Zhihong J, Tong J, Yina L, Zhi Z, Xu Z, Xiaoxing X, Lijuan G. NOX2-mediated reactive oxygen species are double-edged swords in focal cerebral ischemia in mice. J Neuroinflammation. 2022;19(1):184. 35836200 10.1186/s12974-022-02551-6 PMC9281066

[183] Ding Y, Yan Y, Dong Y, Xu J, Su W, Shi W, Zou Q, Yang X. NLRP3 promotes immune escape by regulating immune checkpoints: A pan-cancer analysis. Int Immunopharmacol. 2022;104: Article 108512. 35026655 10.1016/j.intimp.2021.108512

[184] Wang C, Yang T, Xiao J, Xu C, Alippe Y, Sun K, Kanneganti TD, Monahan JB, Abu-Amer Y, Lieberman J, et al. NLRP3 inflammasome activation triggers gasdermin D-independent inflammation. Sci Immunol. 2021;6(64): Article eabj3859. 34678046 10.1126/sciimmunol.abj3859 PMC8780201

[185] de Sa KSG, Amaral LA, Rodrigues TS, Ishimoto AY, de Andrade WAC, de Almeida L, Freitas-Castro F, Batah SS, Oliveira SC, Pastorello MT, et al. Gasdermin-D activation promotes NLRP3 activation and host resistance to *Leishmania* infection. Nat Commun. 2023;14(1):1049. 36828815 10.1038/s41467-023-36626-6 PMC9958042

[186] Du G, Healy LB, David L, Walker C, El-Baba TJ, Lutomski CA, Goh B, Gu B, Pi X, Devant P, et al. ROS-dependent *S*-palmitoylation activates cleaved and intact gasdermin D. Nature. 2024;630(8016):437–446. 38599239 10.1038/s41586-024-07373-5 PMC11283288

[187] Miao N, Kang Z, Wang Z, Yu W, Liu T, Kong LZ, Zheng Y, Ding C, Zhang Z, Zhong C, et al. Mitochondrial reactive oxygen species promote cancer metastasis and tumor microenvironment immunosuppression through gasdermin D. Cell Death Discov. 2025;11(1):219. 40324993 10.1038/s41420-025-02516-7 PMC12053750

[188] Miao C, Huang Y, Zhang C, Wang X, Wang B, Zhou X, Song Y, Wu P, Chen ZS, Feng Y. Post-translational modifications in drug resistance. Drug Resist Updat. 2025;78: Article 101173. 39612546 10.1016/j.drup.2024.101173

[189] Jia H, Jiang L, Shen X, Ye H, Li X, Zhang L, Hu Y, Song D, Jia H, Wang Z. Post-translational modifications of cancer immune checkpoints: Mechanisms and therapeutic strategies. Mol Cancer. 2025;24(1):193. 40629335 10.1186/s12943-025-02397-5 PMC12236040

[190] Ramzan F, Abrar F, Mishra GG, Liao LMQ, Martin DDO. Lost in traffic: Consequences of altered palmitoylation in neurodegeneration. Front Physiol. 2023;14:1166125. 37324388 10.3389/fphys.2023.1166125 PMC10268010

[191] Zmuda F, Chamberlain LH. Regulatory effects of post-translational modifications on zDHHC *S*-acyltransferases. J Biol Chem. 2020;295(43):14640–14652. 32817054 10.1074/jbc.REV120.014717 PMC7586229

[192] Yamaguchi H, Hsu JM, Yang WH, Hung MC. Mechanisms regulating PD-L1 expression in cancers and associated opportunities for novel small-molecule therapeutics. Nat Rev Clin Oncol. 2022;19(5):287–305. 35132224 10.1038/s41571-022-00601-9

[193] Hu X, Lin Z, Wang Z, Zhou Q. Emerging role of PD-L1 modification in cancer immunotherapy. Am J Cancer Res. 2021;11(8):3832–3840. 34522452 PMC8414388

[194] Chen Y, Yue S, Yu L, Cao J, Liu Y, Deng A, Lu Y, Yang J, Li H, Du J, et al. Regulation and function of the cGAS-STING pathway: Mechanisms, posttranslational modifications, and therapeutic potential in immunotherapy. Drug Des Devel Ther. 2025;19:1721–1739. 10.2147/DDDT.S501773 PMC11911240 40098909

[195] Azizi SA, Lan T, Delalande C, Kathayat RS, Banales Mejia F, Qin A, Brookes N, Sandoval PJ, Dickinson BC. Development of an acrylamide-based inhibitor of protein *S*-acylation. ACS Chem Biol. 2021;16(8):1546–1556. 34309372 10.1021/acschembio.1c00405 PMC8590885

[196] Qiu N, Abegg D, Guidi M, Gilmore K, Seeberger PH, Adibekian A. Artemisinin inhibits NRas palmitoylation by targeting the protein acyltransferase ZDHHC6. Cell Chem Biol. 2022;29(3):530–537.e7. 34358442 10.1016/j.chembiol.2021.07.012

[197] Crissey MAS, Versace A, Bhardwaj M, Jain V, Liu S, Singh A, Beer LA, Tang HY, Villanueva J, Gimotty PA, et al. Divergent effects of acute and chronic PPT1 inhibition in melanoma. Autophagy. 2025;21(2):394–406. 39265628 10.1080/15548627.2024.2403152 PMC11760279

[198] Hagenbeek TJ, Zbieg JR, Hafner M, Mroue R, Lacap JA, Sodir NM, Noland CL, Afghani S, Kishore A, Bhat KP, et al. An allosteric pan-TEAD inhibitor blocks oncogenic YAP/TAZ signaling and overcomes KRAS G12C inhibitor resistance. Nat Cancer. 2023;4(6):812–828. 37277530 10.1038/s43018-023-00577-0 PMC10293011

[199] Faergeman NJ, Knudsen J. Role of long-chain fatty acyl-CoA esters in the regulation of metabolism and in cell signalling. Biochem J. 1997;323(Pt 1):1–12. 9173866 10.1042/bj3230001 PMC1218279

[200] Ali A, Levantini E, Teo JT, Goggi J, Clohessy JG, Wu CS, Chen L, Yang H, Krishnan I, Kocher O, et al. Fatty acid synthase mediates EGFR palmitoylation in EGFR mutated non-small cell lung cancer. EMBO Mol Med. 2018;10(3): Article e8313. 29449326 10.15252/emmm.201708313 PMC5840543

[201] Kim YC, Lee SE, Kim SK, Jang HD, Hwang I, Jin S, Hong EB, Jang KS, Kim HS. Toll-like receptor mediated inflammation requires FASN-dependent MYD88 palmitoylation. Nat Chem Biol. 2019;15(9):907–916. 31427815 10.1038/s41589-019-0344-0

[202] Shahid M, Kim M, Jin P, Zhou B, Wang Y, Yang W, You S, Kim J. *S*-palmitoylation as a functional regulator of proteins associated with cisplatin resistance in bladder cancer. Int J Biol Sci. 2020;16(14):2490–2505. 32792852 10.7150/ijbs.45640 PMC7415425

[203] Phan LM, Yeung SC, Lee MH. Cancer metabolic reprogramming: Importance, main features, and potentials for precise targeted anti-cancer therapies. Cancer Biol Med. 2014;11(1):1–19. 24738035 10.7497/j.issn.2095-3941.2014.01.001 PMC3969803

[204] Gansler TS, Hardman W 3rd, Hunt DA, Schaffel S, Hennigar RA. Increased expression of fatty acid synthase (OA-519) in ovarian neoplasms predicts shorter survival. Hum Pathol. 1997;28(6):686–692. 9191002 10.1016/s0046-8177(97)90177-5

[205] Menendez JA, Lupu R. Fatty acid synthase and the lipogenic phenotype in cancer pathogenesis. Nat Rev Cancer. 2007;7(10):763–777. 17882277 10.1038/nrc2222

[206] Guo J, Li N, Liu Q, Hao Z, Zhu G, Wang X, Wang H, Pan Q, Xu B, Han Y, et al. KMT2C deficiency drives transdifferentiation of double-negative prostate cancer and confer resistance to AR-targeted therapy. Cancer Cell. 2025;43(7):1261–1278.e10. 40280125 10.1016/j.ccell.2025.04.002

[207] Sun Y, Zhang H, Meng J, Guo F, Ren D, Wu H, Jin X. S-palmitoylation of PCSK9 induces sorafenib resistance in liver cancer by activating the PI3K/AKT pathway. Cell Rep. 2022;40(7): Article 111194. 35977495 10.1016/j.celrep.2022.111194

[208] Yu Z, Zhang J, Feng J, You G. Palmitoylation transduces the regulation of epidermal growth factor to organic anion transporter 3. Pharmaceutics. 2025;17(7):825. 40733034 10.3390/pharmaceutics17070825 PMC12300882

[209] Rios-Esteves J, Haugen B, Resh MD. Identification of key residues and regions important for porcupine-mediated Wnt acylation. J Biol Chem. 2014;289(24):17009–17019. 24798332 10.1074/jbc.M114.561209 PMC4059143

[210] Buglino JA, Resh MD. Hhat is a palmitoylacyltransferase with specificity for *N*-palmitoylation of Sonic Hedgehog. J Biol Chem. 2008;283(32):22076–22088. 18534984 10.1074/jbc.M803901200 PMC2494920

[211] Zhang Z, Lee YC, Kim SJ, Choi MS, Tsai PC, Xu Y, Xiao YJ, Zhang P, Heffer A, Mukherjee AB. Palmitoyl-protein thioesterase-1 deficiency mediates the activation of the unfolded protein response and neuronal apoptosis in INCL. Hum Mol Genet. 2006;15(2):337–346. 16368712 10.1093/hmg/ddi451

[212] Chan P, Han X, Zheng B, DeRan M, Yu J, Jarugumilli GK, Deng H, Pan D, Luo X, Wu X. Autopalmitoylation of TEAD proteins regulates transcriptional output of the Hippo pathway. Nat Chem Biol. 2016;12(4):282–289. 26900866 10.1038/nchembio.2036 PMC4798901

[213] Yount JS, Moltedo B, Yang YY, Charron G, Moran TM, Lopez CB, Hang HC. Palmitoylome profiling reveals S-palmitoylation-dependent antiviral activity of IFITM3. Nat Chem Biol. 2010;6(8):610–614. 20601941 10.1038/nchembio.405 PMC2928251

[214] Koegl M, Zlatkine P, Ley SC, Courtneidge SA, Magee AI. Palmitoylation of multiple Src-family kinases at a homologous N-terminal motif. Biochem J. 1994;303(3):749–753. 7980442 10.1042/bj3030749 PMC1137610

[215] Galli LM, Anderson MO, Gabriel Fraley J, Sanchez L, Bueno R, Hernandez DN, Maddox EU, Lingappa VR, Burrus LW. Determination of the membrane topology of PORCN, an O-acyl transferase that modifies Wnt signalling proteins. Open Biol. 2021;11(6): Article 200400. 34186010 10.1098/rsob.200400 PMC8241489

[216] Buglino JA, Resh MD. Palmitoylation of Hedgehog proteins. Vitam Horm. 2012;88:229–252. 22391306 10.1016/B978-0-12-394622-5.00010-9 PMC4214369

[217] Shah K, Panchal S, Patel B. Porcupine inhibitors: Novel and emerging anti-cancer therapeutics targeting the Wnt signaling pathway. Pharmacol Res. 2021;167: Article 105532. 33677106 10.1016/j.phrs.2021.105532

[218] Silapunt S, Chen L, Migden MR. Hedgehog pathway inhibition in advanced basal cell carcinoma: Latest evidence and clinical usefulness. Ther Adv Med Oncol. 2016;8(5):375–382. 27583029 10.1177/1758834016653605 PMC4981290

[219] Chen X, Niu W, Fan X, Yang H, Zhao C, Fan J, Yao X, Fang Z. Oct4A palmitoylation modulates tumorigenicity and stemness in human glioblastoma cells. Neuro-Oncology. 2023;25(1):82–96. 35727735 10.1093/neuonc/noac157 PMC9825352

[220] Shao X, Xu A, Du W, Xu T, Huang Y, Xia Z, Wang W, Cai M, Zhang X, Zhang J, et al. The palmitoyltransferase ZDHHC21 regulates oxidative phosphorylation to induce differentiation block and stemness in AML. Blood. 2023;142(4):365–381. 37216691 10.1182/blood.2022019056

[221] Shao XJ, Wang W, Xu AX, Qi XT, Cai MY, Du WX, Cao J, He QJ, Ying MD, Yang B. Palmitoyltransferase ZDHHC3 is essential for the oncogenic activity of PML/RARα in acute promyelocytic leukemia. Acta Pharmacol Sin. 2025;46(2):462–473. 39227737 10.1038/s41401-024-01371-z PMC11747460

[222] Zhang Q, Yang X, Wu J, Ye S, Gong J, Cheng WM, Luo Z, Yu J, Liu Y, Zeng W, et al. Reprogramming of palmitic acid induced by dephosphorylation of ACOX1 promotes β-catenin palmitoylation to drive colorectal cancer progression. Cell Discov. 2023;9(1):26. 36878899 10.1038/s41421-022-00515-x PMC9988979

[223] Chen L, Xing X, Zhu Y, Chen Y, Pei H, Song Q, Li J, Zhang P. Palmitoylation alters LDHA activity and pancreatic cancer response to chemotherapy. Cancer Lett. 2024;587: Article 216696. 38331089 10.1016/j.canlet.2024.216696

[224] Kharbanda A, Walter DM, Gudiel AA, Schek N, Feldser DM, Witze ES. Blocking EGFR palmitoylation suppresses PI3K signaling and mutant KRAS lung tumorigenesis. Sci Signal. 2020;13(621): Article eaax2364. 32127496 10.1126/scisignal.aax2364 PMC7310254

[225] Jennings BC, Nadolski MJ, Ling Y, Baker MB, Harrison ML, Deschenes RJ, Linder ME. 2-Bromopalmitate and 2-(2-hydroxy-5-nitro-benzylidene)-benzo[b]thiophen-3-one inhibit DHHC-mediated palmitoylation in vitro. J Lipid Res. 2009;50(2):233–242. 18827284 10.1194/jlr.M800270-JLR200 PMC2636914

[226] Fan X, Yang H, Zhao C, Hu L, Wang D, Wang R, Fang Z, Chen X. Local anesthetics impair the growth and self-renewal of glioblastoma stem cells by inhibiting ZDHHC15-mediated GP130 palmitoylation. Stem Cell Res Ther. 2021;12(1):107. 33541421 10.1186/s13287-021-02175-2 PMC7863430

[227] Vujic I, Sanlorenzo M, Esteve-Puig R, Vujic M, Kwong A, Tsumura A, Murphy R, Moy A, Posch C, Monshi B, et al. Acyl protein thioesterase 1 and 2 (APT-1, APT-2) inhibitors palmostatin B, ML348 and ML349 have different effects on NRAS mutant melanoma cells. Oncotarget. 2016;7(6):7297–7306. 26771141 10.18632/oncotarget.6907 PMC4872786

[228] Xu J, Hedberg C, Dekker FJ, Li Q, Haigis KM, Hwang E, Waldmann H, Shannon K. Inhibiting the palmitoylation/depalmitoylation cycle selectively reduces the growth of hematopoietic cells expressing oncogenic Nras. Blood. 2012;119(4):1032–1035. 22144181 10.1182/blood-2011-06-358960 PMC3271715

[229] Virlogeux A, Scaramuzzino C, Lenoir S, Carpentier R, Louessard M, Genoux A, Lino P, Hinckelmann MV, Perrier AL, Humbert S, et al. Increasing brain palmitoylation rescues behavior and neuropathology in Huntington disease mice. Sci Adv. 2021;7(14): Article eabb0799. 33789888 10.1126/sciadv.abb0799 PMC8011966

[230] Zhuang Z, Gu J, Li BO, Yang L. Inhibition of gasdermin D palmitoylation by disulfiram is crucial for the treatment of myocardial infarction. Transl Res. 2024;264:66–75. 37769810 10.1016/j.trsl.2023.09.007

[231] Chen S, Han C, Miao X, Li X, Yin C, Zou J, Liu M, Li S, Stawski L, Zhu B, et al. Targeting MC1R depalmitoylation to prevent melanomagenesis in redheads. Nat Commun. 2019;10(1):877. 30787281 10.1038/s41467-019-08691-3 PMC6382811

[232] Brun S, Bestion E, Raymond E, Bassissi F, Jilkova ZM, Mezouar S, Rachid M, Novello M, Tracz J, Hamai A, et al. GNS561, a clinical-stage PPT1 inhibitor, is efficient against hepatocellular carcinoma via modulation of lysosomal functions. Autophagy. 2022;18(3):678–694. 34740311 10.1080/15548627.2021.1988357 PMC9037544

[233] Rebecca VW, Nicastri MC, McLaughlin N, Fennelly C, McAfee Q, Ronghe A, Nofal M, Lim CY, Witze E, Chude CI, et al. A unified approach to targeting the lysosome’s degradative and growth signaling roles. Cancer Discov. 2017;7(11):1266–1283. 28899863 10.1158/2159-8290.CD-17-0741 PMC5833978

[234] Liu J, Pan S, Hsieh MH, Ng N, Sun F, Wang T, Kasibhatla S, Schuller AG, Li AG, Cheng D, et al. Targeting Wnt-driven cancer through the inhibition of porcupine by LGK974. Proc Natl Acad Sci USA. 2013;110(50):20224–20229. 24277854 10.1073/pnas.1314239110 PMC3864356

[235] Bollu LR, Katreddy RR, Blessing AM, Pham N, Zheng B, Wu X, Weihua Z. Intracellular activation of EGFR by fatty acid synthase dependent palmitoylation. Oncotarget. 2015;6(33):34992–35003. 26378037 10.18632/oncotarget.5252 PMC4741504

[236] Haag SM, Gulen MF, Reymond L, Gibelin A, Abrami L, Decout A, Heymann M, van der Goot FG, Turcatti G, Behrendt R, et al. Targeting STING with covalent small-molecule inhibitors. Nature. 2018;559(7713):269–273. 29973723 10.1038/s41586-018-0287-8

[237] Holden JK, Crawford JJ, Noland CL, Schmidt S, Zbieg JR, Lacap JA, Zang R, Miller GM, Zhang Y, Beroza P, et al. Small molecule dysregulation of TEAD Lipidation induces a dominant-negative inhibition of Hippo pathway signaling. Cell Rep. 2020;31(12): Article 107809. 32579935 10.1016/j.celrep.2020.107809

[238] Xiong W, Sun KY, Zhu Y, Zhang X, Zhou YH, Zou X. Metformin alleviates inflammation through suppressing FASN-dependent palmitoylation of Akt. Cell Death Dis. 2021;12(10):934. 34642298 10.1038/s41419-021-04235-0 PMC8511025

[239] Gridnev A, Maity S, Misra JR. Structure-based discovery of a novel small-molecule inhibitor of TEAD palmitoylation with anticancer activity. Front Oncol. 2022;12:1021823. 36523977 10.3389/fonc.2022.1021823 PMC9745137

[240] Hu L, Sun Y, Liu S, Erb H, Singh A, Mao J, Luo X, Wu X. Discovery of a new class of reversible TEA domain transcription factor inhibitors with a novel binding mode. eLife. 2022;11: Article e80210. 36398861 10.7554/eLife.80210 PMC9728997

[241] Kong Y, Liu Y, Li X, Rao M, Li D, Ruan X, Li S, Jiang Z, Zhang Q. Palmitoylation landscapes across human cancers reveal a role of palmitoylation in tumorigenesis. J Transl Med. 2023;21(1):826. 37978524 10.1186/s12967-023-04611-8 PMC10655258

[242] Wu W, Hu L. inventors; General Hospital Corp, assignee. Small molecule inhibitors of TEAD–YAP. US patent US20240279191A1. 2024 August 22.

[243] Patterson SI, Skene JH. Novel inhibitory action of tunicamycin homologues suggests a role for dynamic protein fatty acylation in growth cone-mediated neurite extension. J Cell Biol. 1994;124(4):521–536. 8106550 10.1083/jcb.124.4.521 PMC2119910

